# Neuroprotective Strategies after Neonatal Hypoxic Ischemic Encephalopathy

**DOI:** 10.3390/ijms160922368

**Published:** 2015-09-15

**Authors:** Brandon J. Dixon, Cesar Reis, Wing Mann Ho, Jiping Tang, John H. Zhang

**Affiliations:** 1 Department of Physiology and Pharmacology, Loma Linda University School of Medicine, Loma Linda, CA 92354, USA; E-Mails: bjdixon@llu.edu (B.J.D.); reis.cesar.llu@gmail.com (C.R.); wingiho@googlemail.com (W.M.H.); Jtang@llu.edu (J.T.); 2 Department of Anesthesiology, Loma Linda University Medical Center, Loma Linda, CA 92354, USA; 3 Department of Neurosurgery, Medical University Innsbruck, Tyrol 6020, Austria; 4 Department of Neurosurgery, Loma Linda University School of Medicine, Loma Linda, CA 92354, USA

**Keywords:** neonatal hypoxic ischemic encephalopathy, neuroprotection, intervention strategy, therapeutic strategy

## Abstract

Neonatal hypoxic ischemic encephalopathy (HIE) is a devastating disease that primarily causes neuronal and white matter injury and is among the leading cause of death among infants. Currently there are no well-established treatments; thus, it is important to understand the pathophysiology of the disease and elucidate complications that are creating a gap between basic science and clinical translation. In the development of neuroprotective strategies and translation of experimental results in HIE, there are many limitations and challenges to master based on an appropriate study design, drug delivery properties, dosage, and use in neonates. We will identify understudied targets after HIE, as well as neuroprotective molecules that bring hope to future treatments such as melatonin, topiramate, xenon, interferon-beta, stem cell transplantation. This review will also discuss some of the most recent trials being conducted in the clinical setting and evaluate what directions are needed in the future.

## 1. Introduction

### 1.1. Neonatal Hypoxic Ischemic Encephalopathy

Neonatal hypoxic ischemic encephalopathy (HIE) is a devastating disease that primarily causes neuronal and white matter injury. HIE has tremendous detrimental effects on the developing brain and is among the leading causes of death among infants, as well as the major underlying cause of seizures in term infants [1,2,3]. Although there have been major advances in modern technology and an increased understanding of fetal and neonatal pathologies, HIE is still a serious condition that is unresolved and causes significant mortality and long-term morbidity [4,5,6,7].

Neonatal HIE can also be characterized as an injury that occurs in the immature brain, resulting in delayed cell death via excitotoxicity, inflammation, and oxidative stress [4]. These adverse events in the developing brain often lead to long lasting detrimental neurological defects later on in life such as mental retardation, epilepsy, cerebral palsy, learning disabilities, and other neurophysiological handicaps [8]. Care for newborn infants at risk for hypoxia ischemia is a priority in health care and understanding the pathophysiology of hypoxic ischemic brain injury is quite essential to the design of effective interventions [9].

Before the advent of hypothermia, clinicians were not able to provide much care to neonates suffering from HIE besides systemic supportive care [10]. It is necessary to explicate interventions that will rid young children’s lives of this form of stroke. Thus, this review aims to characterize the current pathophysiology of HIE, and describe and elucidate complications that are creating a gap between basic science and clinical translation. In addition, we aim to analyze promising neuroprotective strategies after HIE and prognosticate how treatment strategies will change and what new therapeutic strategies are on the horizon.

### 1.2. Incidence and Prognosis of HIE

Neonatal hypoxic ischemic encephalopathy is of great importance since it is the major cause and contributor to global infant mortality and morbidity [11]. The incidence of neonatal HIE in the United States is 2–3 in 1000 live births, with evidence of incidences being up to 6 births in 1000 live births [11]. Underdeveloped countries have even reported incidences up to 26 per 1000 live births [10]. About 20%–25% of term newborn infants die during the neonatal period and about 25% of those that survive develop permanent neurological disabilities. Patients with mild grades of encephalopathy are generally reported to have normal cognitive functions by school age. While patients with moderate grading are associated with a spectrum of long-term disabilities and significant motor and cognitive disabilities [10]. 

### 1.3. Clinical Presentation

Neonatal hypoxic ischemic encephalopathy usually presents clinically in the earliest days of life in a term infant and can be characterized by difficulty initiating and maintaining respiration, depression of tone and reflexes, subnormal levels of consciousness, and multiple seizures [10].

Since HIE is the major underlying cause of seizures in term infants, a large variety of seizures may present [12]. Subtle, clonic, tonic, myoclonic that are focal, multifocal, or generalized are the most common types of seizures that present [13,14,15]. In the perinatal period hypoxemia, ischemia, and other impairments to the exchange of respiratory gases often give rise to asphyxia. Thus, neonatal HIE is described as acute intrapartum events that cause moderate to severe neonatal encephalopathy, metabolic acidosis in fetal umbilical arterial blood obtained at delivery, spastic or dyskinetic quadraparesis, and absence of other causes of cerebral palsy [16]. It is also critically important to eliminate other causes of neonatal encephalopathy in order to prevent delay in diagnosis and neuroprotective intervention [17].

In order to classify neonatal HIE and the degree of injury, the Sarnat 3 stage grading system is used and widely accepted. This system consists of three stages ranging from mild, moderate, and severe, all based on clinical symptoms described above, along with electroencephalogram evaluation [18].

### 1.4. Current Therapeutic Strategies

Presently, there are no well-established effective therapies for neonatal HIE [19]. Hypothermia is a method of protection that is used to treat full term neonates with moderate to severe HIE. Along with hypothermia, comprehensive clinical care of mechanical ventilation, physiological and biochemical monitoring, neuroimaging, seizure detection and monitoring, and neurological consultation are also included. Although hypothermia does provide some protection, only 1 in 6 infants benefit from hypothermia [20]. There is an area of uncertainty involving hypothermia in regards to depth and duration of cooling, and information involving premature infants born less than 35 weeks of gestational age. Thus, it is critical to have an appropriate treatment to provide protection to neonates suffering from HIE [21].

## 2. Potential Intervention Targets

**Figure 1:**
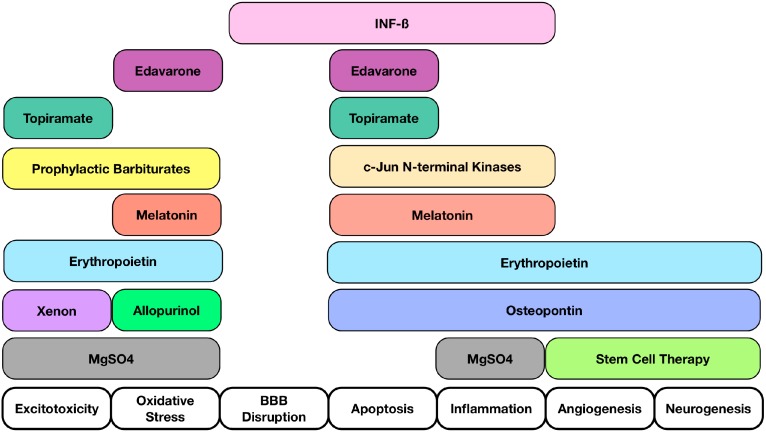
Interventional Targets Following Hypoxic Ischemic Encephalopathy (HIE). A summary of potential interventional targets following HIE along with molecules that can exert multiple properties. Interventional targets consist of Excitotoxicity, Oxidative Stress, Blood Brain Barrier Disruption, Apoptosis, Inflammation, Angiogenesis, and Neurogenesis. Multiple target sites suggest that a multi-targeted approach is beneficial after HIE.

Since the pathophysiology of HIE injury is quite complex, there are a myriad of potential interventional targets following HIE where prevention of cellular damage can occur [22] (Figure 1). Targeting impaired function sites like the neurovascular unit, attempting to quell apoptosis, inflammation, or promoting neurogenesis and angiogenesis are all strategic points of importance [10,23]. Although targets such as apoptosis or necrosis have been previously explored, there are other areas of importance that have been understudied [24,25]. We will now delve into the pathophysiology and discuss some promising understudied intervention sites.

### 2.1. Pathophysiology

The pathophysiology involving neonatal HIE consists of multiple phases [10]. One the first phases that results is changes in the vasculature. If placental blood flow is disrupted a period of asphyxia occurs, causing a loss in auto-regulation and the development of cerebral blood flow being dependent on systemic arterial pressure. As a result of this change in regulation of cerebral blood flow; a decrease in systemic arterial blood pressure increases the risk for tissue acidosis and ischemic brain injury [10,26].

**Figure 2:**
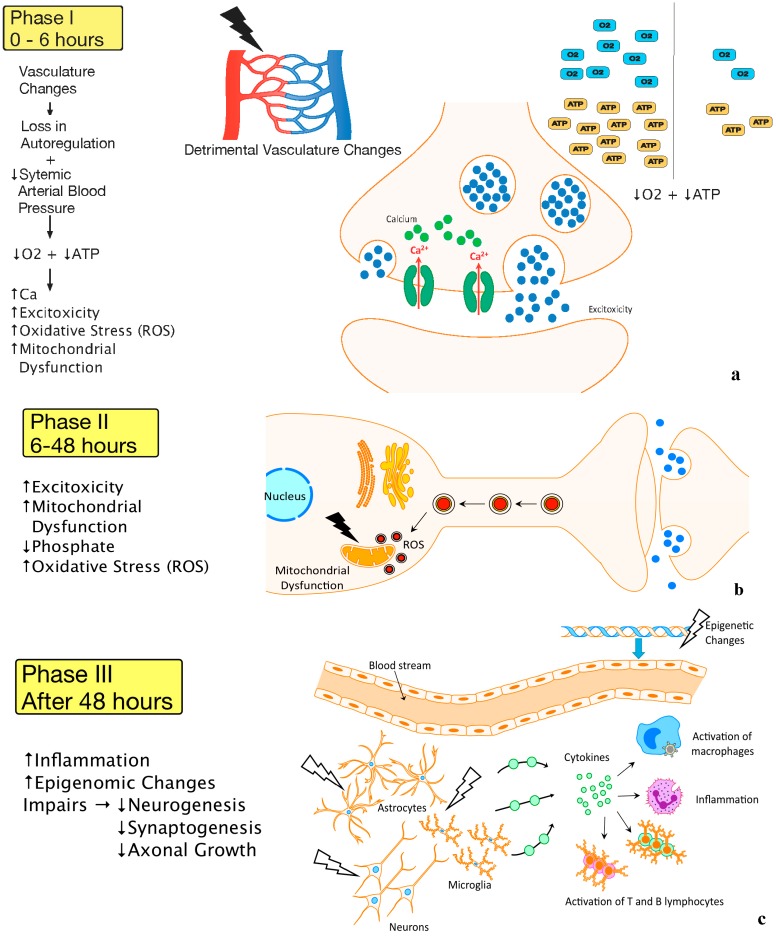
(**a**) Vasculature Changes and Primary Energy Failure (Phase I) Legend: A visual representation of the first phase of HIE. Detrimental changes to the vasculature following an HIE insult lead to loss of autoregulation and severe lowering of the systemic arterial blood pressure. This causes a decrease in oxygen, depletion of ATP, as well as increases in excitotoxicity, intracellular calcium, oxidative stress, and mitochondrial dysfunction; (**b**) Secondary Energy Failure (Phase II). Legend: A schematic representation of the second phase of HIE reveals continued excitotoxicity, oxidative stress, and mitochondrial dysfunction; (**c**) Chronic Inflammation (Phase III). A pictorial representation of the third phase of HIE shows injury to microglia, neurons, and astrocytes leads to continuous release of cytokines and other detrimental factors causing chronic inflammation which in turn leads to epigenetic changes, as well as impairments of synaptogenesis, axonal growth, and neurogenesis.

The next phase that follows is a primary energy failure phase that occurs at the cellular level (Figure 2a). Since there is a loss of oxygen that is readily available to the brain, cellular energy metabolism shifts to a dependency upon anaerobic metabolism. This reliance upon anaerobic metabolism pathways leads to the collection of lactic acid and depletion of adenosine triphosphate (ATP) [27]. The loss of cellular homeostasis also leads to an intracellular accumulation of sodium, calcium, water, and excitatory neurotransmitter release causing an “excitotoxic-oxidative cascade”. Increasing cellular influx of calcium also occurs as a consequence of excessive stimulation of neurotransmitter receptors and membrane depolarization [28,29]. Further influx of calcium also leads to increased activation of lipase, causing a release of fatty acids, and increased activation of neuronal nitric oxide synthase giving rise to free radical production and mitochondrial dysfunction [26]. As a consequence, mitochondrial dysfunction ultimately signals pathways of apoptotic or necrotic cell death. Apoptotic cell death is believed to occur when energy supplies are not completely exhausted, while necrotic cell death occurs when energy supplies are no longer available [27].

A second energy failure phase also occurs 6–48 h after an episode of hypoxia ischemia [30] (Figure 2b). The second energy failure phase also results in the detrimental release of excitatory neurotransmitters and free radicals as well as depletion of high phosphate reserves, but differs from the primary energy failure phase since it is independent of cerebral acidosis [30,31].

A third phase where deleterious factors cause further damage and potentiates injury and worsens outcomes has recently been proposed (Figure 2c). This third phase is thought to include mechanisms of inflammation and epigenetic changes that lead to an impairment or alteration of axonal growth, neurogenesis, and synaptogenesis [29].

### 2.2. Blood-Brain Barrier

The neurovascular unit, also referred to as the blood-brain barrier (BBB), consists of the basement membrane, capillary endothelial cells, tight junctions, pericytes, and astrocytes [32]. It is believed that all the components that comprise the BBB are important for stability and proper functioning [33]. Thus, injury to the cerebrovasculature and the BBB after HIE leads to detrimental effects since it is an essential diffusion barrier required for normal functioning of the central nervous system [33]. In an observational study of term neonates with HIE, it was found that BBB permeability is a contributing factor to HIE [34]. In order to assess permeability of the BBB in this study, the cerebrospinal fluid albumin to plasma albumin ratio was measured in 43 HIE neonates and was compared with 20 normal gestational age and gender-matched healthy infants without HIE. The results showed that plasma albumin levels were similar in neonates with and without HIE. However, cerebral spinal fluid (CSF) albumin levels were 5 times higher in infants with HIE when compared with controls indicating permeability of the BBB [32,34,35].

It is believed that cytokines, nitric oxide, and vascular endothelial growth factor (VEGF) help regulate mechanisms that lead to tight junction disruption and increased BBB permeability [33]. Increased levels of proinflammatory cytokines have also been observed in animal brains after global and focal ischemia as well as in cerebrospinal fluid of stroke patients [33].

After HIE it is postulated that there are biphasic temporal patterns of BBB opening. Numerous *in vitro* and *in vivo* studies have revealed that VEGF and matrix metalloproteinases (MMP) are involved in the initial opening of the BBB within hours of an hypoxic-ischemic event [32]. While the second opening, occurring around 6–24 h, involves the activation of microglia and astrocytes which release proinflammatory cytokines that induces MMPs and cyclooxygenases triggering protease destruction and reactive oxygen species (ROS) production respectively [32]. Once the barrier is disrupted, detrimental events can be further exacerbated by the infiltration of neutrophils and monocytes from the systemic compartment.

Although many adverse outcomes occur once the BBB is permeable, there are experimental studies that show that stabilizing the BBB and reducing inflammatory markers after hypoxic ischemic events as well as HIE may be beneficial [36,37,38,39,40]. For example, a study exploring the mechanisms of granulocyte-colony stimulating factor (G-CSF) as a treatment after a neonatal rat model of HIE, demonstrated that G-CSF elicited BBB stabilization by G-CSF receptor stimulation and activation of the phosphoinositide 3-kinase (PI3K)/Akt pathway. The enhancement of the BBB occurred in endothelial cells through increases in tight junction proteins, claudin 3 and claudin 5, in addition to decreases in vascular cell adhesion protein 1 (VCam-1) and intercellular adhesion molecule 1 (ICam-1) adherens proteins [41].

Similarly another study utilizing a neonatal rat model administered plasminogen activator inhibitor-1 following an HIE insult. This group was able to demonstrate that inhibition of tissue plasminogen activator (tPA) was able to prevent MMP activation, prevent HIE-tPA induced opening of the BBB, and decrease tPA-converted plasmin activities as a protease that normally degrades the extracellular matrix and the BBB [42].

Some strides have been made, yet further investigations are needed to better understand BBB integrity after HIE [35]. It has been suggested that endothelial cells may be more susceptible to oxygen deprivation, while astrocytes and pericytes are tolerant of sole oxygen deprivation, indicating that a more complete understanding of the cellular relationship that comprises the BBB may be critical to formulating strategies of protection following hypoxic ischemic events [43]. Also, more recent knowledge seems to indicate that the BBB may be more stable during the developmental period than maturation [35]. Thus, any and all information regarding the mechanisms of BBB formation and disruption will help in constructing strategies of BBB protection. 

### 2.3. Angiogenesis

Angiogenesis is characterized as the process involving the growth of new vessels that extend the existing blood circulation into avascular regions. The angiogenesis process consists of vascular basal lamina formation, proliferation and migration, and tubular formation of migrating cells. Some findings suggest that angiogenesis is activated after acute insult in the neonatal brain [44]. One study comparing asphyxiated newborns without brain injury, asphyxiated newborns with brain injury, and healthy newborns discovered that angiogenesis pathways maybe dysregulated after HIE. This dysregulation was evidenced by the observation that asphyxiated newborns with brain injury had decreased expression of insulin-growth factor binding protein-1, -4, and -6, which are anti-angiogenic proteins. While asphyxiated newborns that did not develop brain injury showed increases in fatty acid binding protein 4, glucose-6-phosphate isomerase, expression of neuropilin-1 (a vascular endothelial growth factor receptor-2 co-receptor), and receptor tyrosine-protein kinase erbB-3, which are proteins associated with endothelial cell survival, proliferation, and migration [45]. Thus, angiogenesis seems to be a viable target in newborns with HIE [44]. In the adult brain, angiogenesis is a key repair mechanism after a hypoxic ischemic event. However, several factors concerning angiogenesis remains to be elucidated after injury in term newborns [44]. Further studies to discover how angiogenesis can be activated to enhance brain repair after HIE in newborns are essential to creating viable strategies on how to improve functional and structural recovery after injury [44]. One study indicated that angiogenesis is activated after HIE injury by observing hyperfusion measured by magnetic resonance imaging (MRI) during the first month [44]. It has also been shown that angiogenesis could be induced by the transplantation of CD34+ cells from umbilical cord blood and that this improvement is beneficial [46]. Together the data suggests that the neovascularization mechanisms are essential for survival and may be a potential therapeutic site after stroke.

### 2.4. Neurogenesis

Neurogenesis is comprised of cell proliferation, migration and differentiation [47]. It is believed that neurogenesis continues even throughout adulthood. The site for neurogenesis occurs in the subventricualar zone and subgranular layer of the hippocampal dentate gyrus, where the local environment tightly regulates neurogenesis. Although there is evidence that suggests that neurogenesis increases after injuries such as HIE, the endogenous repair mechanisms do not resolve the brain damage that occurs [48,49,50]. Ong *et al.* observed increases of doublecortin, a microtubule-associated protein only expressed in immature neurons, with immunostaining in the subventricular zone ipsilateral to HIE-induced lesioning one to three weeks post injury. However, after the fourth week there were no identifiable newly formed mature striatal neurons, suggesting that there was limited neurogenesis at this time period. These results also suggest depletion of the neuronal progenitor pool since they are more vulnerable to HIE than SVZ stem cells or that the environment is not conducive to the maturation and survival of newly formed neurons since there maybe a lack of trophic support [51]. Thus, aiding or boosting the endogenous repair mechanisms after brain injuries may restore function. Therefore, understanding the molecular and cellular mechanism of neurogenesis and whether or not these mechanisms can be clinically applicable presents neurogenesis as a prime therapeutic target [50,51].

There have been a number of promising stem cell therapy experiments that promote neurogenesis after injury. For example, van Velthoven *et al.* demonstrated that bone marrow derived mesenchymal stem cells treatment after HIE was able to increase neurogenesis and formation of new oligodendrocytes, evidenced by NeuN-positive and olig2-positive cells [52]. Again, it is quite important that more studies are conducted to optimize and elucidate mechanisms following injury. Discovering and understanding the factors that promote neurogenesis during normal physiological conditions as well as the impairment that occurs during brain damage are key questions that need to be determined [50,53].

### 2.5. Autophagy

After HIE, there is evidence of differential cellular death mechanisms often occurring in different cells. One cellular death mechanism that has increased scientific interests and has been recently implicated in HIE has a potential therapeutic target is autophagy [24,54]. Autophagy is a lysosomal pathway for intracellular degradation of macromolecules and organelles that plays an important part to maintaining cellular survival and homeostasis [55]. Since autophagy is a tightly regulated process, controversy of whether or not autophagy provides beneficial effects after HIE currently exists [56]. There are some experiments that describe pharmacological inhibition of autophagy being neuroprotective, while others have shown that inducing autophagy immediately after injury may be an endogenous neuroprotective mechanism [4].

For example, Chen *et al.* demonstrated that brain-derived neurotrophic factor (BDNF) with a concentration range of 50 to 200 ng/mL protects neurons from hypoxia injury *in vitro* via the induction of autophagy [57]. It was shown that BDNF could induce autophagy through the PI3K/Akt/mammalian target of rapamycin (mTOR) pathway. In this study, activation of mTOR complex 1 lead to phosphorylation of ribosomal protein S6 kinase (p70S6K), a controller of protein translation, which ultimately induced autophagy and exerted protective effects [57]. Conversely, a study evaluating the effects of lithium in an *in vivo* model of neonatal HIE brain injury found that lithium was able to inhibit post-ischemic autophagy after 72 h. This reduction of autophagy was indicated by decreased number of LC3-positive cells in lithium-treated animals 72 h post-HIE. Given these results, it is unclear if a reduction of autophagy occurred as a secondary effect to lithium induced neuroprotection since there is less cellular debris and damage contributing to autophagy processing or if lithium directly inhibited autophagy [55]. Mixed results involving autophagy suggests that deleterious or neuroprotective effects depend upon specific regions, the severity of the insult, and the timing of activation [4]. Over the past decades, much has been learned about autophagy; however, more studies to elucidate exact mechanisms remain, especially following neurodegenerative insults and diseases [54].

## 3. Limitations to Intervention Sites

### 3.1. Limitations to in Vitro and in Vivo

The distinct mechanisms of injury-induced damage in immature brain is frequently studied, yet remains poorly understood, especially at early stages of development. Also, a thorough evaluation method of the developmental and behavioral sequelae after brain injury needs to be established [58].

In general, there are *in vitro* and *in vivo* models of experimental studies. *In-vitro* studies are relatively inexpensive and often precede a following *in vivo* analysis, yet it has its limitations of treating cells outside of their normal environment. Therefore, their results may differ from *in vivo* effects [59]. If *in vivo* tests can confirm *in vitro* results, the informative value of the results is more significant. In most cases, drug studies proven to be effective *in vitro*, show ineffective in animal models. The underlying reason may be drug delivery issues, toxicity, or other problems [60] Even though several reviews demonstrated poorly predictive value of animal models on human outcomes, those experiments are mandatory especially in drug development before translation to clinical trials [61,62,63].

### 3.2. Limitations to Animal Models

Especially since HIE and neonatal stroke are very heterogeneous entities, therefore finding the ideal method to study this disorder remains a difficult task [64]. Ashwal and colleagues compared the HIE model by Rice-Vannucci and the neonatal stroke filament occlusion model using MRI. Obviously, the stroke model injury was defined on the ipsilateral middle cerebral artery territory, while more generalized and greater cortical ischemia occurred with the Rice-Vannucci method, even involving the contralateral side in some cases [65].

Other new promising neonatal hypoxic-ischemic models have been introduced recently, like a perinatal global ischemic brain injury model in rats providing intrapartum hypoxia in rats [66]. Another perinatal method accomplished systemic hypoxic-ischemic brain injury by cutting off the placenta blood supply to the fetuses to mimic brain damage in early preterm newborns [67]. One model of postnatal permanent occlusion of the middle cerebral artery was described using direct electrocoagulation aiming for a highly reproducible, more consistent, and selective cortical infarction area [68].

The great variety of established models and promising new designs points out the importance of carefully choosing the appropriate model, especially for testing possible treatment strategies [69]. A few key points may be that the timing and nature of the induced ischemic injuries vary tremendously in the different models. Additionally, the translation from rodent studies to human trials are discussed broadly, because of diverse and in some extent lacking neurological responses to sensorimotor cortex lesions when compared to humans. This may demand non-human primate experiments for more neurological similarity [70]. Thus, only one perinatal hypoxic-ischemic brain injury model in primates has been developed so far, showing similar anatomical and cellular pathology in cortex development, as in post-ischemic observations in newborns [71]. Although, biologically proximity to humans seems to be important in animal studies, over 95% of experiments in general were performed in rats and mice [72,73]. Other points to consider are the inevitable delays between symptom onset and start of treatment [74,75] or the outcome assessment within days in animal models in contrast to after months in patients [76].

Although much progress in experimental development and technology has been made in the past few decades, the issue of discovering disease models, that can mimic human pathophysiology sufficiently, remains an ongoing challenge [73,77,78].

### 3.3. Limitations to Central Nervous System Drug Delivery

Treatment strategies for central nervous system (CNS) diseases are challenged by the drug delivery mechanisms for crossing the BBB [79]. The underlying reason, also considered the bottleneck in CNS drug development, is in most cases since insufficient attention to the prediction and assessment of the compounds ability to cross the BBB [80]. Without solving the BBB problem, CNS drugs are limited to lipid-soluble and low molecular weight (less than 400 Daltons) compounds. Progress in molecular neuroscience might therefore lead to more availability of effective CNS therapeutics [81]. This demands research focusing on signaling pathways and trafficking mechanisms, as involved brain transporters at the BBB and brain-cerebrospinal fluid-barrier [82].

In a recent publication, rapid and transient BBB disruptions were described after HIE in neonatal mice associated with alteration of tight-junction proteins. This response is often followed by activation of tightening compensatory mechanisms. More understanding of this phenomenon might provide an opportunity window for compounds to access the brain tissue easier after HIE [83].

### 3.4. Limitations to Neonatal Drugs and Dosage

Additionally, in cases of neonatal HIE the clinical translation is more difficult when compared to adult drug administrations, since most of the pharmacological developments are approved only for adults and the regulations for pediatric and neonatal use are highly restricted. Due to the small size and huge variability of pediatric patients, neonatal pharmacotherapies are limited to mostly off label usage of compounds developed for adults to extrapolate dosages [84,85,86]. Another issue besides the small number yet huge variety of patients challenging clinical trials and regulations are possible side effects, as in adverse drug reactions in the individualized neonatal treatment strategies [87].

Progressive research in neonates might improve and optimize future age-appropriate treatment demanding tailored, personalized clinical pharmacological and physiological understanding [29,88,89,90]. Further research topics involve drug excipients for analyzing and initiating the safety and toxicity for pediatrics (STEP) database and the European Study of Neonatal Excipient Exposure (ESNEE) initiative [91,92,93].

## 4. Potential Novel Molecules and Strategies for Neuroprotection after HIE

Over the past decade, clinical and basic science research in neonates has achieved huge progress in neuroprotective and neurointensive care, and established hypothermia as the standard of care treatment for neonatal HIE. Although there has been much progress, further investigation is needed to discover adjuvant neuroprotective strategies [94]. This is also important for designing clinical and experimental studies since therapeutic targets might alternate in the phases of HIE, in addition to the physiological development changes that occurs in neonates over time [29]. We will now discuss some molecules that are neuroprotective and are currently being evaluated in experimental translational studies (Table 1) and clinical trials (Table 2) for therapeutic potential after HIE. 

**Table 1:** Neuroprotective molecules evaluated in experimental translational studies after HIE. Summary of neuroprotective molecules used in experimental translational studies and their proposed mechanisms of action associated with HIE.

Molecules Studied	Possible Effects Related to Neuroprotection
Osteopontin (OPN)	OPN repairs brain injury after neonatal HIE by mediating regulation of cerebral cell proliferation, cell survival, and oligodendrocyte differentiation after injury [95]
Interferon Beta (INFβ)	Reduce TNF-α levels, proliferation and activation of T-cell lymphocytes, and pro-inflammatory cytokines produced by T-cells; Blood Brain Barrier integrity [96]
c-Jun N-terminal kinases (JNKs)	JNKs play a role in regulation of apoptosis [97]; Reductions in early neuronal damage [98]; Reduced inflammation and inhibition of apoptotic neuronal loss [99]
Prophylactic barbiturates	Diminishes moderate to severe neurodevelopmental impairment or death (HIE undergoing whole-body cooling) [100]; Multivariate analysis suggested its use to be associated with better outcomes [100]
Melatonin	Antioxidant, anti-inflammatory, and anti-apoptotic properties [101,102]; Protect the brain independently or in concert with therapeutic hypothermia [103]; Reducing oxidative stress and improved survival with favorable neurodevelopmental outcome at 6 months of age in combination with hypothermia [104]
Edaravone	Edaravone may inhibit the number of apoptotic neuronal cells and 8-OHdG expression within 48 h after HI insult [105]; Inhibits lipid peroxidation in neonatal HIE rat model [106]; Scavenger that inhibits both lipid and DNA peroxidation [107]

**Table 2:** Neuroprotective molecules evaluated in current clinical trials involving HIE. Summary of neuroprotective molecules that are currently being studied in HIE clinical trials and their proposed mechanisms of action.

Molecules Studied	Possible Effects Related to Neuroprotection
Erythropoietin (EPO)	Associated with anti-inflammatory, anti-excitotoxic, anti-oxidative, and anti-apoptotic properties [108,109,110]; Vasogenic and pro-angiogenic functions [109]; Hypoxia-inducible-factor-1 mediates increase in EPO expression [111,112]
Allopurinol	Neuroprotection in postnatal day 7 rats after HI [113]; *Post hoc* analysis revealed a potential benefit in treatment of females [114]
Xenon	May trigger neurodegeneration in the developing brain. Thus, the safety of a newborn injured brain is not expected [115]; Neuroprotective in adult rats in transient brain ischemia [115]; Limited protection when given alone but protection for up to 30 days when given in combination with hypothermia (neonatal rodents) [116]
Topiramate (TPM)	AMPA and Kainate receptors inhibition [117,118,119]; Blockade of Na channels, high voltage-activated calcium currents, carbonic anhydrase isoenzymes and mitochondrial permeability transition pore [120,121,122,123]; TPM in concert with melatonin decreases infarcted volume and apoptosis in neonatal HI rat model [124]; Pretreatment significantly reduced the brain damage and subsequent cognitive impairments [125]
Magnesium Sulfate (MgSO_4_)	Controversies exist regarding its efficacy in protecting the brain in term infants who may suffer encephalopathy [126]
Cord blood	Rich with hematopoietic stem cells [127,128,129,130,131] and neurotrophic factors acting on the following: Immunomodulation, reduction of immune cell infiltration, and the potential to increase neurogenesis and an angiogenesis [132,133]

### 4.1. Experimental Translational Studies

#### 4.1.1. Osteopontin

Osteopontin (OPN) is a mutifunctional glycoprotein with increased upregulation in the brain after neonatal HIE. OPN has both pro- and anti- inflammatory properties; thus, its exact role in injury is not well predicted [95,134,135]. OPN repairs brain injury after neonatal HIE by mediating regulation of cerebral cell proliferation, cell survival, and oligodendrocyte differentiation after injury [136]. According to Chen and colleagues, OPN-induced neuroprotection was associated with cleaved caspase-3 inhibition and anti-apoptotic cell death, thereby improving long-term neurological function against neonatal HIE brain injury [137]. However, a recent study done by Bonestroo and colleagues demonstrated that intranasal, intraperitoneal (i.p.), and intracerebral administration of a small TAT-OPN peptide was neither neuroprotective by measuring the anatomical reduction of the HIE induced brain injury, nor beneficial in reducing sensorimotor behavioral deficits [138]. Endogenous expression of OPN was shown to be highest in the brain at age 0 with continuous reductions until day 21 during development. After HIE injury, endogenous OPN expression increased and peaked at 48 h. Exogenous OPN decreased infarct volume and improved neurological outcomes 7 weeks after HIE injury [137].

#### 4.1.2. Interferon Beta

Inflammation plays an important role in the pathology of HIE, interventions at the inflammation portion of the disease can potentially be beneficial [19]. Thus, it is plausible that the positive immunomodulatory effects of interferon beta (IFNβ) as seen in an inflammatory environment such as multiple sclerosis (MS) will also have a therapeutic effect in the neonatal HIE model. In experimental models of MS, IFNβ has been shown to reduce tumor necrosis factor alpha (TNF-α) levels, proliferation and activation of T-cell lymphocytes, and pro-inflammatory cytokines produced by T-cells [96]. Intrastriatal injections IFNβ has been shown to preserve the BBB integrity, decrease infarct size, and block the infiltration of inflammatory cells in a middle cerebral artery occlusion model [139]. In a model of transient focal stroke, it was reported that intravenous tail injections of IFNβ failed to provide protection. It appears that IFNβ is unable to cross BBB; thus, methods to circumvent the BBB are needed in order for IFNβ to be effective [140]. 

#### 4.1.3. c-Jun N-Terminal Kinases

c-Jun N-terminal kinases (JNKs) activation is associated with an assortment of environmental stressors and for that reason they are known as stress activated protein kinases [141,142]. Through phosphorylation and modification of proteins residing in the mitochondria, JNKs play a role in regulation of apoptosis [97]. Nijboer and colleagues demonstrated reductions in early neuronal damage in P7 rats at 0 and 3 h after HIE [98]. They injected intraperitoneally TAT-JBD, a JNK inhibitor, in a neonatal model of HIE brain injury. Post insult administration reduced brain damage and lasted up to 14 weeks post-HIE. Furthermore, sensory, cognitive, and behavioral benefits were associated with the 50% anatomical cerebral improvements found in their study. These results indicated that the activity of JNK in the brain was inhibited effectively by TAT-JBD treatment [143]. In 2013 Nijboer and colleagues also demonstrated that inhibition of phosphorylation of mitochondrial JNK may lead to preventing early loss of mitochondrial integrity, consequently leading to reduced inflammation and inhibition of apoptotic neuronal loss. Up-regulation of anti-apoptotic mitochondrial proteins also played a crucial role in maintaining neuroprotection [99].

The TAT-JBD peptide may serve as a treatment option for neonatal HIE due to its promising results in reducing neuronal damage and loss of mitochondrial activity through early JNK inhibition, with overall improvements in anatomical outcomes, therefore improving cognitive and behavioral results post-HIE. The present study shows that early JNK inhibition by the short-lived TAT-JBD peptide may be a promising therapy for neonatal HIE by conferring long-term anatomical and behavioral improvements [143].

#### 4.1.4. Prophylactic Barbiturates

A retrospective study by Donald F. Meyn, Jr. and colleagues [100] analyzed the effects of prophylactic administration of phenobarbital to infants with HIE. They found that phenobarbital administration to infants with HIE undergoing whole-body cooling diminishes moderate to severe neurodevelopmental impairment or death. Despite their small sample size, they found that this combination diminishes clinically detectable seizures. On the other hand, their study failed to improve neurodevelopmental outcomes significantly by univariate analysis. However, multivariate analysis suggested its use to be associated with better outcomes. Though the most effective dose, most effective timing of administration, and the most effective drug are not known, the treatment combination used by Meyn Jr. and colleagues may help disrupt the cascade of injury.

Therapeutic interventions enabling prevention or reduction in hypoxia-induced brain damage before or during an earlier stage of free-radical production will require continued investigation for optimal effectiveness [144]. These results and findings set the stage for a large and multicenter randomized-control trial that includes a long term follow up analysis to further test the incremental benefit of prophylactic anticonvulsant therapy in the setting of hypothermia [100]. 

#### 4.1.5. Melatonin

Melatonin (*N*-acetyl-5-methoxytryptamine) is an endogenous indolamine and another scavenger that has shown promising effects in the treatment of HIE. It has antioxidant, anti-inflammatory, and anti-apoptotic properties [101]. Melatonin freely crosses the placenta and the blood-brain barrier making it an attractive agent for neuroprotection. In an asphyxia animal model it has been shown to protect the brain independently [102] or in concert with therapeutic hypothermia [103]. Aly and colleagues demonstrated that the combination of melatonin and therapeutic hypothermia in infants with moderate to severe HIE was efficacious in reducing oxidative stress and improved survival with favorable neurodevelopmental outcome at 6 months of age [104]. Intravenous use of melatonin showed efficacy and feasibility when used in neonates with HIE who were receiving whole body therapeutic hypothermia [104]. With its effectiveness for both pre-term and term infants [145] it holds considerable promise as an adjunct therapy [146] and results from various studies suggest combination therapy as the most effective. Optimal dose, route, and duration of administration are still parameters that need to be researched in depth in order to help in clinical translation.

#### 4.1.6. Edaravone

Edaravone (3-methyl-1-phenyl-2-pyrazolin-5-one) is a free scavenger that is thought to be useful for the treatment of acute cerebral stroke. Edaravone is believed to interact with peroxyl and hydroxyl radicals creating a radical intermediate that forms stable oxidation products [105,106]. Ni X. and colleagues also demonstrated the benefits of edaravone as an antioxidant agent in HIE. They observed that systemic administration of edaravone 30 min after resuscitation from HIE can salvage neurons in the striatum in a large animal model of neonatal HIE [107]. Furthermore another study showed that intraperitoneal administration of edaravone after HIE for consecutive days improved memory and learning ability when given in the acute phase of HIE [147].

### 4.2. Current Clinical Trial Studies

#### 4.2.1. Erythropoietin

Erythropoietin (Epo) is a 34 kilodalton glycoprotein with pleotropic properties. Epo has been reported to have effects on a variety of receptor-mediated and cell-specific mechanisms that are beneficial and essential after HIE. Epo has been associated with anti-inflammatory, anti-excitotoxic, anti-oxidative, and anti-apoptotic effects as well as promoting neurogenesis and angiogenesis [108,109,110]. Epo is expressed in both human and animal brains in its early development but decreases gradually after birth [148].

Hypoxia-inducible factor-1 mediates the increase of Epo after HIE in the brain, leading not only to an increase in Epo expression but also an increase in the Epo receptor in neurons, astrocytes, and microglia [111,112]. Epo levels are increased in newborn infants with HIE in the cerebrospinal fluid despite the absence of exogenous Epo treatment [148]. In the setting of HIE there is an increase in permeability of the blood brain barrier [149,150,151], allowing high doses of Epo to increase its levels in the CSF [149,150,152,153]. Studies have shown that neonatal rats with HIE injuries have histological and functional improvements following high-doses of Epo and that multiple doses reduces infarct volumes in a dose-dependent manner [154]. Kumral *et al.* demonstrated that a single dose of Epo (1000 U/kg i.p.) immediately after neonatal hypoxic–ischemic insult diminished long-term spatial memory deficits. In addition, a treatment group that received Epo but did not undergo HIE, showed no differential effects concerning learning or memory from the treatment [155]. One clinical study also showed that Epo 1000 and 2500 U/kg per dose intravenously administered along with hypothermia achieved and surpassed plasma concentrations that provided neuroprotection in animal models [154]. Furthermore a study evaluating middle cerebral artery occlusion in neonatal rats found that three daily doses of Epo (1 U/g, i.p. each) caused increases in hemispheric volume and its sub regions, as well as spatial learning and memory [156].

There have been several completed clinical trials concerning Epo after HIE injury [150,157,158,159]. Currently there are two active clinical trials (NCT01913340 and NCT01732146) examining Epo in combination with hypothermia in infants with HIE. The “Neonatal Erythropoietin And Therapeutic Hypothermia Outcomes in Newborn Brain Injury” study (NCT01913340) assesses an Epo dose of 1000 U/kg/dose IV × 5 doses. While the “Efficacy of Erythropoietin to Improve Survival and Neurological Outcome in Hypoxic Ischemic Encephalopathy” study (NCT01732146) evaluates Epo intravenous injections (5000 U/0.3 mL) 1000 to 1500 U/kg/dose three times given every 24 h with the first dose within 12 h of delivery.

Epo exerts neuroprotection through phosphorylation of its receptor and Janus Kinase 2, which provides a docking complex for intracellular signaling proteins including PI3K as well as Akt, signal transducer and activator of transcription 5 (STAT5), and the extracellular signal-regulated kinase (ERK). Activating these pathways leads to alteration of cell proliferation, survival, and differentiation by affecting a number of downstream targets. For example, Akt limits inflammation [160] and decreases apoptotic cell death. STAT5 acts on cell survival [161], while the ERK pathway has demonstrated not only to have anti-apoptotic and anti-inflammatory effects *in vitro* but also to be essential for neurogenesis and cell fate commitment [162,163].

#### 4.2.2. Allopurinol

Allopurinol is a xanthine oxidase inhibitor that lowers uric acid concentration in patients with gout and neoplastic diseases. In addition, allopurinol functions as a chelator of non-protein bound iron as well as a direct scavenger of hydroxyl radicals, suggesting it may serve in neuroprotection [164]. Indeed, allopurinol provided neuroprotection in postnatal day 7 rats after HIE [113]. Even though Benders and colleagues did not show improvement in outcomes with after-birth asphyxia [165], a recent follow-up study in human neonates with allopurinol used on term asphyxiated neonates showed benefits on mortality and severe disabilities at 4–8 years of age [166]. In addition, Kaandorp and colleagues investigated the pharmacological applicability of allopurinol for intrauterine neuroprotection after maternal administration. They showed intravenously administered allopurinol to the mother rapidly crosses the placenta with satisfactory concentrations reaching the neonate at birth. In addition, it is safe to both the mother and the neonate.

In 2014, the same group published the results of another clinical trial. Maternal treatment with allopurinol during fetal hypoxia did not significantly lower neuronal damage markers in umbilical cord blood. However, *post hoc* analysis revealed a potential benefit in treatment of females (NCT00189007) [114]. There is an ongoing study investigating the reduction in free radical formation after reperfusion with initiation of this medication during labor, with the intention of reducing free radical induced post asphyxia brain damage. They hope to demonstrate how allopurinol during asphyxia reduces post-hypoxic-ischemic reperfusion damage in the newborn (NCT00189007). In addition, there is another ongoing trial (the European ALBINO trial) that will assess outcomes at 2 years of life. Studies have shown allopurinol is a viable treatment option for early fetal neuroprotective therapy during labor, but future studies and clinical investigation are necessary to further support its effectiveness.

#### 4.2.3. Xenon

Xenon is a potent anesthetic with a low gas partition coefficient. It crosses the BBB easily and guarantees rapid induction of anesthesia. As an anesthetic it has proven to be safe in adults and well tolerated [167]. However, a recent review by Istaphanous and Loepke demonstrated that xenon may trigger neurodegeneration in the developing brain [168]. Thus, the safety of a newborn injured brain is not expected.

Important neuroprotective effects of Xenon have been demonstrated in adult rats in transient brain ischemia [115]. In neonatal rodents, it was associated with relatively limited protection when given alone but did protect for up to 30 days when given in combination with hypothermia [116]. Similarly, other studies have also proven this combination to be beneficial [169,170].

Xenon was reported to be safe for use in a phase II randomized study outcomes after demonstrating to have similar results as cooling therapy alone [171]. An ongoing clinical trial (NCT01545271), estimated to be completed in October 2015, aims to examine the effect of inhaled xenon gas in the treatment of newborn infants with HIE in combination with cooling, which is the standard treatment of this condition. They hypothesize that the xenon and cooling combination will produce better neuroprotection than the standard treatment of cooling alone. Hypothermia plus adjuvant therapies have been extensively reviewed in two recent publications [172,173]. Based on the preclinical studies, ongoing trials in neonates include inhaled Xenon and cooling (NCT01545271 and NCT00934700).

#### 4.2.4. Topiramate 

Topiramate (TPM) is an anticonvulsant agent with multiple mechanisms of action [174,175], implying its ability to be a neuroprotective agent. It has neuroprotective qualities according to previous literature. Its neuroprotective mechanisms appear to be related not only to AMPA and Kainate receptors inhibition [117,118,119,176,177] but also to blockade of Na^+^ channels [120], high voltage-activated calcium currents [121], carbonic anhydrase isoenzymes [122], and mitochondrial permeability transition pore [123].

Even though no clinical studies have been published to prove an additive or synergistic action of TPM in concert with hypothermia in newborns, ongoing clinical trials (NCT01765218), Topiramate in Neonates Receiving Whole Body Cooling for Hypoxic Ischemic Encephalopathy, are investigating whether topiramate improves the outcomes of babies with neonatal hypoxic encephalopathy who are receiving whole body cooling. This trial is to be completed in 2017.

TPM in concert with melatonin decreases infarcted volume and apoptosis in neonatal HIE rat model [124]. In addition, Noh and colleagues [125] reported that i.p. or per oral topiramate pretreatment significantly reduced the brain damage and subsequent cognitive impairments induced by hypoxia-ischemia in neonatal rats. Similarly, it leads to dose dependent and long lasting neuroprotection in the excitotoxic newborn mouse model [177]. Topiramate is able to provide neuroprotection by increasing survival of pre-oligodendrocytes, decreasing neuronal apoptosis, inhibiting microglial activation and astrogliosis, and decreasing seizure activity.

Melatonin and topiramate, acting on different stages of HIE, used alone or in combination, significantly decreased the percent infarcted area, and apoptotic cell death in neonatal HIE rat model. It is necessary to investigate different doses and application times of these agents as combination therapy in order to provide more effective neuroprotection. Furthermore, an ongoing trial: The NeoNATI trial (NCT01241019) will evaluate neurological outcomes at 6, 12, and 18 months of life and help clarify questions as to whether the administration of TPM in newborns with HIE potentiates the neuroprotective effect of treatment with hypothermia. They hypothesize that the combination treatment with moderate whole-body hypothermia associated with TPM administration is safe and enhances the neuroprotective properties of hypothermia for the treatment of neonatal HIE

#### 4.2.5. Magnesium Sulfate 

Magnesium Sulfate (MgSO_4_) has gained a lot of interest in the research community due to its ability to alleviate excitotoxic damage *in vitro* by binding to the magnesium site on *N*-methyl-d-aspartate (NMDA) glutamate channel [178]. Evidence leads researchers to believe that it also reduces secondary inflammation and associated injury [179], acts on cell membrane stabilization and inhibition of free radical production [180], and improves cardiovascular stability [181].

MgSO_4_ is also known to be neuroprotective. However, controversy regarding its efficacy in protecting the brain in term infants who may suffer encephalopathy exists. These thoughts emerged due to the fact that the outcomes of previous studies are highly inconsistent when it comes to neuroprotection. Differences in dose and timing of administration were present amidst evidence of beneficial effects [126].

Robert Galinsky and colleagues showed that the effect of MgSO_4_ treatment before or shortly after acute HIE at term or near-term equivalent was highly inconsistent between studies [126]. This caused questions and concerns to arise regarding the benefits of MgSO_4_ since the perinatal studies on this topic did not directly control brain or body temperature, yet suggested beneficial effects of MgSO_4_. In addition, most of these rodent studies didn’t control environmental temperatures. The studies in which the body temperature was controlled in large animal translational models suggested lack of effect after 2 or 3 days of recovery [182,183,184].

Tagin and colleagues also demonstrated that there is insufficient evidence to determine if magnesium therapy given shortly after birth to newborns with HIE reduces death or moderate-to-severe disability [127]. Currently, an ongoing phase III clinical trial (NCT01646619) is assessing whether the addition of a drug such as MgSO_4_ while providing therapeutic hypothermia or cooling to babies who are asphyxiated at birth provides additional benefit to the survival and outcomes compared to cooling alone. Severe Neurodevelopmental Disability will be assessed at discharge from the hospital and at 18–24 months of age to assess developmental delay and cerebral palsy.

There is insufficient evidence to determine if magnesium therapy given shortly after birth to newborns with HIE reduces death or moderate to severe disability. The improvement in short-term outcomes without significant increase in adverse effects supports the need for further adequately powered trials to determine if there are long-term benefits of magnesium and to confirm its safety. Mortality should be monitored closely in all future trials involving magnesium therapy for newborns with HIE. In the current review, the results, although statistically insignificant for mortality between the magnesium and the control groups, showed a trend toward an increase in mortality in the magnesium group.

#### 4.2.6. Stem Cell Therapy and Neonatal HIE

**Figure 3:**
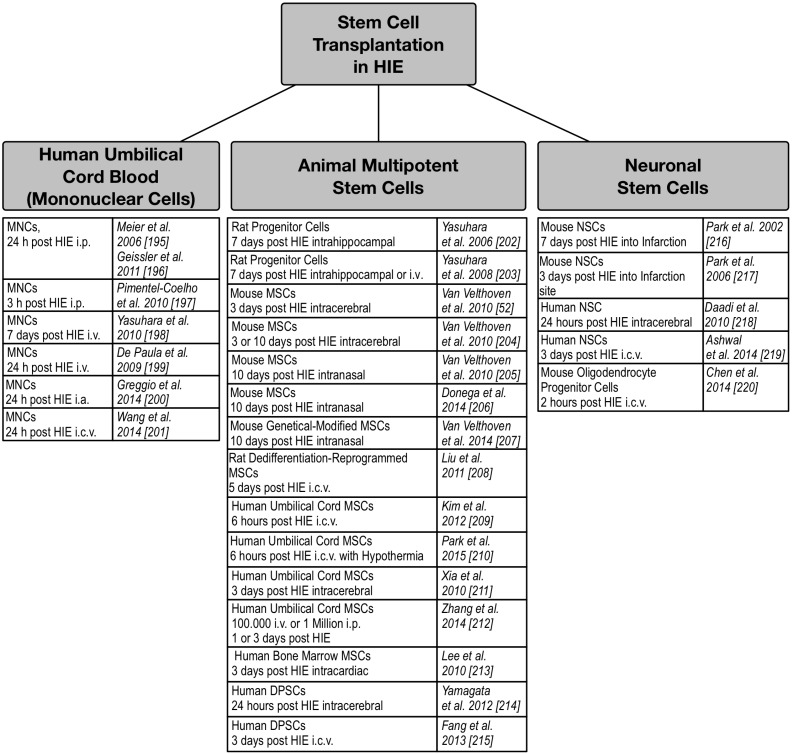
Stem Cell Transplantation in Animal Models of HIE. Summary of stem cell transplantation studies in various animal models of HIE. Human Dental Pulp Stem Cells (DPSCs); Hypoxic Ischemic Encephalopathy (HIE); Mononuclear Cells (MNCs); Mesenchymal Stem Cells (MSCs); Neuronal Stem Cells (NSC); Based upon cell dose, cell type, transplantation timing, and administration route.

Stem cell therapy represents a modern cornerstone of promising neuroprotective and neuroregenerative treatment options that can benefit from ongoing trials, especially in adult stroke [128]. However, in context of perinatal HIE, it has gained importance as adjunct treatment with hypothermia in recent clinical trials to meliorate mortality and chronic neurological disability. Several sources for stem cells include neural stem/progenitor cells derived from fetal tissue, mesenchymal stem cells or embryonic stem-induced pluripotent stem cells [129,130] (Figure 3).

Additionally, cord blood (CB) represents a rich source of stem cells used in several animal models of neurological diseases [129,130,131,132,185]. Autologous transplantation of CB, collected shortly after delivery, has the advantages of minimal *ex vivo* manipulation, no necessary immunosuppression, relatively easy access, and storage properties. CB is rich in primitive stem cells, yet it contains a limited number of cell types, mostly mononuclear cells, and showed to be not as pluripotent as embryonic stem cells [133]. Studies analyzing the risk and benefits of autologous CB infusion in neonates with HIE and in children with cerebral palsy show promising results [133,186,187]. Placebo-controlled clinical trials are demanded. Currently ongoing clinical trials include the initiated trial by Cotten *et al.* of autologous CB infusion in term infants with HIE (NCT00593242) [133,188].

Due to the lack of imaging diagnostic difficulties to detect HIE in premature newborns and insufficient data, present stem cell therapy trials are restricted to full term infants [189,190]. Further investigation is needed for developing the best strategy considering transplantation timing, cell dosage, *ex vivo* modulations, way of administration, and choice of stem cells [133]. It should be mentioned that, besides stem cell transplantation, there is research ongoing in the field of stem cell factors. G-CSF [3], and glial-cell derived neurotrophic factor have shown promising results [191].

**Figure 4:**
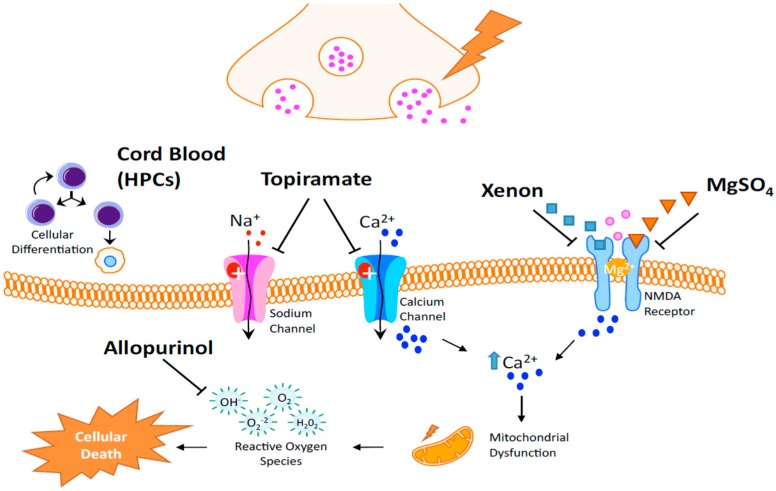
Proposed Mechanisms of Current Clinical Trials. Cord blood infusions are rich with hematopoietic stem cells and neurotrophic factors that have numerous effects such as immunomodulation, reduction of microglia and T-lymphocyte infiltration, as well as the potential to increase neurogenesis and an angiogenesis. It is believed that topiramate is able to block sodium channels and high voltage-activated calcium currents after HIE. Xenon is believed to bind at the glycine site of the NMDA receptor and inhibit its downstream effects. Similarly magnesium sulfate also inhibits the NMDA receptor by binding to the magnesium site of the receptor. Allopurinol is predicted to provide neuroprotection by directly scavenging hydroxyl radicals after HIE injury.

As reviewed above, the complex etiology of HIE requires treatment that will act on multiple processes [192]. There is an important unmet need to further improve the outcome of neonatal encephalopathy in term infants. The agents mentioned in this section either alone or in combination deserve rigorous and focused testing in order to render better results that would allow researchers to translate the studies to clinical scenarios. Optimal dose, route, and duration of administration are still parameters that need to be researched in depth in order to provide better guidance about the next step to follow. Any favorable results might lead to new perspectives leading to reduction of cerebral damage in asphyxiated newborns. Intensive tests are needed to provide a platform for furthering clinical trials to better support their use in the clinical setting and answering many questions that remain to be answered (Figure 4).

## 5. Conclusions and Future Perspectives

Although there have been significant strides in the basic sciences to create novel neuroprotective and therapeutic strategies to combat HIE, there is still much more research needed to be conducted in order to translate potential therapies. Some gaps in our knowledge concerning the pathophysiology and the timing of important endogenous neuroprotective and neuroregenerative mechanisms still exist. In order to make basic science results more clinically relevant and translational, combinational therapies with hypothermia should be considered and studied. There is also a need for more biomarker studies that can be used along with the brain imaging, and long-term neuro-assessments [193,194]. Thus, the field of neonatal HIE has many promising avenues and possibilities in the realm of translational research. 

## References

[1] Shetty J. (2015). Neonatal seizures in hypoxic-ischaemic encephalopathy-risks and benefits of anticonvulsant therapy. Dev. Med. Child. Neurol..

[2] Volpe J.J. (2001). Perinatal brain injury: From pathogenesis to neuroprotection. Ment. Retard. Dev. Disabil. Res. Rev..

[3] Doycheva D., Shih G., Chen H., Applegate R., Zhang J.H., Tang J. (2013). Granulocyte-colony stimulating factor in combination with stem cell factor confers greater neuroprotection after hypoxic-ischemic brain damage in the neonatal rats than a solitary treatment. Transl. Stroke Res..

[4] Northington F.J., Chavez-Valdez R., Martin L.J. (2011). Neuronal cell death in neonatal hypoxia-ischemia. Ann. Neurol..

[5] Gill M.B., Perez-Polo J.R. (2008). Hypoxia ischemia-mediated cell death in neonatal rat brain. Neurochem. Res..

[6] Badr Zahr L.K., Purdy I. (2006). Brain injury in the infant: The old, the new, and the uncertain. J. Perinat. Neonatal Nurs..

[7] Shankaran S. (2012). Hypoxic-ischemic encephalopathy and novel strategies for neuroprotection. Clin. Perinat..

[8] Fathali N., Lekic T., Zhang J.H., Tang J. (2010). Long-term evaluation of granulocyte-colony stimulating factor on hypoxic-ischemic brain damage in infant rats. Intensive Care Med..

[9] Wayock C.P., Meserole R.L., Saria S., Jennings J.M., Huisman T.A., Northington F.J., Graham E.M. (2014). Perinatal risk factors for severe injury in neonates treated with whole-body hypothermia for encephalopathy. Am. J. Obstet. Gynecol..

[10] Douglas-Escobar M., Weiss M.D. (2015). Hypoxic-ischemic encephalopathy: A review for the clinician. JAMA Pediatr..

[11] Logitharajah P., Rutherford M.A., Cowan F.M. (2009). Hypoxic-ischemic encephalopathy in preterm infants: Antecedent factors, brain imaging, and outcome. Pediatr. Res..

[12] Vasudevan C., Levene M. (2013). Epidemiology and aetiology of neonatal seizures. Semin. Fetal Neonatal Med..

[13] Lombroso C.T. (2007). Neonatal seizures: Gaps between the laboratory and the clinic. Epilepsia.

[14] Sheth R.D. (1999). Electroencephalogram confirmatory rate in neonatal seizures. Pediatr. Neurol..

[15] Silverstein F.S., Jensen F.E. (2007). Neonatal seizures. Ann. Neurol..

[16] Eunson P. (2015). The long-term health, social, and financial burden of hypoxic-ischaemic encephalopathy. Dev. Med. Child. Neurol..

[17] Volpe J.J. (2012). Neonatal encephalopathy: An inadequate term for hypoxic-ischemic encephalopathy. Ann. Neurol..

[18] Robertson C.M., Perlman M. (2006). Follow-up of the term infant after hypoxic-ischemic encephalopathy. Paediatr. Child. Health.

[19] Fathali N., Khatibi N.H., Ostrowski R.P., Zhang J.H. (2011). The evolving landscape of neuroinflammation after neonatal hypoxia-ischemia. Acta Neurochir. Suppl..

[20] Wachtel E.V., Hendricks-Munoz K.D. (2011). Current management of the infant who presents with neonatal encephalopathy. Curr. Probl. Pediatr. Adolesc. Health Care.

[21] Yager J.Y., Ashwal S. (2009). Animal models of perinatal hypoxic-ischemic brain damage. Pediatr. Neurol..

[22] Allen K.A., Brandon D.H. (2011). Hypoxic ischemic encephalopathy: Pathophysiology and experimental treatments. Newborn Infant Nurs. Rev..

[23] Baburamani A.A., Ek C.J., Walker D.W., Castillo-Melendez M. (2012). Vulnerability of the developing brain to hypoxic-ischemic damage: Contribution of the cerebral vasculature to injury and repair?. Front. Physiol..

[24] Ginet V., Puyal J., Clarke P.G., Truttmann A.C. (2009). Enhancement of autophagic flux after neonatal cerebral hypoxia-ischemia and its region-specific relationship to apoptotic mechanisms. Am. J. Pathol..

[25] Seevinck P.R., Deddens L.H., Dijkhuizen R.M. (2010). Magnetic resonance imaging of brain angiogenesis after stroke. Angiogenesis.

[26] Johnston M.V., Trescher W.H., Ishida A., Nakajima W. (2001). Neurobiology of hypoxic-ischemic injury in the developing brain. Pediatr. Res..

[27] Lai M.C., Yang S.N. (2011). Perinatal hypoxic-ischemic encephalopathy. J. Biomed. Biotechnol..

[28] Kumar P., Halamek L.P. (2012). Resuscitation of the Fetus and Newborn, an Issue of Clinics in Perinatology.

[29] Juul S.E., Ferriero D.M. (2014). Pharmacologic neuroprotective strategies in neonatal brain injury. Clin. Perinatol..

[30] Lorek A., Takei Y., Cady E.B., Wyatt J.S., Penrice J., Edwards A.D., Peebles D., Wylezinska M., Owen-Reece H., Kirkbride V. (1994). Delayed ("secondary") cerebral energy failure after acute hypoxia-ischemia in the newborn piglet: Continuous 48-hour studies by phosphorus magnetic resonance spectroscopy. Pediatr. Res..

[31] Vannucci R.C., Towfighi J., Vannucci S.J. (2004). Secondary energy failure after cerebral hypoxia-ischemia in the immature rat. J. Cereb. Blood Flow Metab..

[32] Laptook A. (2014). The importance of temperature on the neurovascular unit. Early Hum. Dev..

[33] Ballabh P., Braun A., Nedergaard M. (2004). The blood-brain barrier: An overview: Structure, regulation, and clinical implications. Neurobiol. Dis..

[34] Kumar A., Mittal R., Khanna H.D., Basu S. (2008). Free radical injury and blood-brain barrier permeability in hypoxic-ischemic encephalopathy. Pediatrics.

[35] Moretti R., Pansiot J., Bettati D., Strazielle N., Ghersi-Egea J.F., Damante G., Fleiss B., Titomanlio L., Gressens P. (2015). Blood-brain barrier dysfunction in disorders of the developing brain. Front. Neurosci..

[36] Wu J., Zhao D., Wu S., Wang D. (2015). Ang-(1–7) exerts protective role in blood-brain barrier damage by the balance of timp-1/mmp-9. Eur. J. Pharmacol..

[37] Zhao T., Zhang X., Zhao Y., Zhang L., Bai X., Zhang J., Zhao X., Chen L., Wang L., Cui L. (2014). Pretreatment by evodiamine is neuroprotective in cerebral ischemia: Up-regulated pakt, pgsk3beta, down-regulated nf-kappab expression, and ameliorated bbb permeability. Neurochem. Res..

[38] Hou C.W., Chen Y.L., Chuang S.H., Wang J.S., Jeng K.C. (2014). Protective effect of a sesamin derivative, 3-bis (3-methoxybenzyl) butane-1, 4-diol on ischemic and hypoxic neuronal injury. J. Biomed. Sci..

[39] Wang Y.F., Gu Y.T., Qin G.H., Zhong L., Meng Y.N. (2013). Curcumin ameliorates the permeability of the blood-brain barrier during hypoxia by upregulating heme oxygenase-1 expression in brain microvascular endothelial cells. J. Mol. Neurosci..

[40] Song J., Cheon S.Y., Lee W.T., Park K.A., Lee J.E. (2015). The effect of ask1 on vascular permeability and edema formation in cerebral ischemia. Brain Res..

[41] Li L., McBride D.W., Doycheva D., Dixon B.J., Krafft P.R., Zhang J.H., Tang J. (2015). G-csf attenuates neuroinflammation and stabilizes the blood-brain barrier via the pi3k/akt/gsk-3beta signaling pathway following neonatal hypoxia-ischemia in rats. Exp. Neurol..

[42] Yang D., Nemkul N., Shereen A., Jone A., Dunn R.S., Lawrence D.A., Lindquist D., Kuan C.Y. (2009). Therapeutic administration of plasminogen activator inhibitor-1 prevents hypoxic-ischemic brain injury in newborns. J. Neurosci..

[43] Engelhardt S., Huang S.F., Patkar S., Gassmann M., Ogunshola O.O. (2015). Differential responses of blood-brain barrier associated cells to hypoxia and ischemia: A comparative study. Fluids Barriers CNS.

[44] Shaikh H., Lechpammer M., Jensen F.E., Warfield S.K., Hansen A.H., Kosaras B., Shevell M., Wintermark P. (2015). Increased brain perfusion persists over the first month of life in term asphyxiated newborns treated with hypothermia: Does it reflect activated angiogenesis?. Transl. Stroke Res..

[45] Shaikh H., Boudes E., Khoja Z., Shevell M., Wintermark P. (2015). Angiogenesis dysregulation in term asphyxiated newborns treated with hypothermia. PLoS ONE.

[46] Pimentel-Coelho P.M., Rosado-de-Castro P.H., da Fonseca L.M., Mendez-Otero R. (2012). Umbilical cord blood mononuclear cell transplantation for neonatal hypoxic-ischemic encephalopathy. Pediatr. Res..

[47] Chen A., Xiong L.J., Tong Y., Mao M. (2013). The neuroprotective roles of bdnf in hypoxic ischemic brain injury. Biomed. Rep..

[48] Eriksson P.S., Perfilieva E., Bjork-Eriksson T., Alborn A.M., Nordborg C., Peterson D.A., Gage F.H. (1998). Neurogenesis in the adult human hippocampus. Nat. Med..

[49] Guidi S., Bianchi P., Alstrup A.K., Henningsen K., Smith D.F., Bartesaghi R. (2011). Postnatal neurogenesis in the hippocampal dentate gyrus and subventricular zone of the gottingen minipig. Brain Res. Bull..

[50] Donega V., van Velthoven C.T., Nijboer C.H., Kavelaars A., Heijnen C.J. (2013). The endogenous regenerative capacity of the damaged newborn brain: Boosting neurogenesis with mesenchymal stem cell treatment. J. Cereb. Blood Flow Metab..

[51] Ong J., Plane J.M., Parent J.M., Silverstein F.S. (2005). Hypoxic-ischemic injury stimulates subventricular zone proliferation and neurogenesis in the neonatal rat. Pediatr. Res..

[52] Van Velthoven C.T., Kavelaars A., van Bel F., Heijnen C.J. (2010). Mesenchymal stem cell treatment after neonatal hypoxic-ischemic brain injury improves behavioral outcome and induces neuronal and oligodendrocyte regeneration. Brain Behav. Immun..

[53] Fernandez-Lopez D., Natarajan N., Ashwal S., Vexler Z.S. (2014). Mechanisms of perinatal arterial ischemic stroke. J. Cereb. Blood Flow Metab..

[54] Ohsumi Y. (2014). Historical landmarks of autophagy research. Cell. Res..

[55] Li Q., Li H., Roughton K., Wang X., Kroemer G., Blomgren K., Zhu C. (2010). Lithium reduces apoptosis and autophagy after neonatal hypoxia-ischemia. Cell. Death Dis..

[56] Balduini W., Carloni S., Buonocore G. (2012). Autophagy in hypoxia-ischemia induced brain injury. J. Mater. Fetal Neonatal Med..

[57] Chen A., Xiong L.J., Tong Y., Mao M. (2013). Neuroprotective effect of brain-derived neurotrophic factor mediated by autophagy through the pi3k/akt/mtor pathway. Mol. Med. Rep..

[58] Vannucci S.J., Hagberg H. (2004). Hypoxia-ischemia in the immature brain. J. Exp. Biol..

[59] Hartung T., Daston G. (2009). Are *in vitro* tests suitable for regulatory use?. Toxicol. Sci..

[60] De Clercq E. (2005). Recent highlights in the development of new antiviral drugs. Curr. Opin. Microbiol..

[61] Perel P., Roberts I., Sena E., Wheble P., Briscoe C., Sandercock P., Macleod M., Mignini L.E., Jayaram P., Khan K.S. (2007). Comparison of treatment effects between animal experiments and clinical trials: Systematic review. BMJ.

[62] Hackam D.G., Redelmeier D.A. (2006). Translation of research evidence from animals to humans. JAMA.

[63] O'Collins V.E., Macleod M.R., Donnan G.A., Horky L.L., van der Worp B.H., Howells D.W. (2006). 1026 experimental treatments in acute stroke. Ann. Neurol..

[64] Johnston M.V., Ferriero D.M., Vannucci S.J., Hagberg H. (2005). Models of cerebral palsy: Which ones are best?. J. Child. Neurol..

[65] Ashwal S., Tone B., Tian H.R., Chong S., Obenaus A. (2007). Comparison of two neonatal ischemic injury models using magnetic resonance imaging. Pediatr. Res..

[66] Yang T., Zhuang L., Terrando N., Wu X., Jonhson M.R., Maze M., Ma D. (2011). A clinically relevant model of perinatal global ischemic brain damage in rats. Brain Res..

[67] Huang Y., Lai H., Xu H., Wu W., Lai X., Ho G., Ma L., Chen Y. (2013). Impact of perinatal systemic hypoxic-ischemic injury on the brain of male offspring rats: An improved model of neonatal hypoxic-ischemic encephalopathy in early preterm newborns. PLoS ONE.

[68] Tsuji M., Ohshima M., Taguchi A., Kasahara Y., Ikeda T., Matsuyama T. (2013). A novel reproducible model of neonatal stroke in mice: Comparison with a hypoxia-ischemia model. Exp. Neurol..

[69] Northington F.J. (2006). Brief update on animal models of hypoxic-ischemic encephalopathy and neonatal stroke. ILAR J..

[70] Clowry G.J., Basuodan R., Chan F. (2014). What are the best animal models for testing early intervention in cerebral palsy?. Front. Neurol..

[71] Teo L., Bourne J.A. (2014). A reproducible and translatable model of focal ischemia in the visual cortex of infant and adult marmoset monkeys. Brain Pathol..

[72] Sena E., van der Worp H.B., Howells D., Macleod M. (2007). How can we improve the pre-clinical development of drugs for stroke?. Trends Neurosci..

[73] Van der Worp H.B., Howells D.W., Sena E.S., Porritt M.J., Rewell S., O'Collins V., Macleod M.R. (2010). Can animal models of disease reliably inform human studies?. PLoS Med..

[74] Saver J.L., Johnston K.C., Homer D., Wityk R., Koroshetz W., Truskowski L.L., Haley E.C. (1999). Infarct volume as a surrogate or auxiliary outcome measure in ischemic stroke clinical trials. Stroke.

[75] The National Institute of Neurological Disorders and Stroke (Ninds) rt-pa Stroke Study Group (2000). Effect of intravenous recombinant tissue plasminogen activator on ischemic stroke lesion size measured by computed tomography. Stroke.

[76] Van der Worp H.B., de Haan P., Morrema E., Kalkman C.J. (2005). Methodological quality of animal studies on neuroprotection in focal cerebral ischaemia. J. Neurol..

[77] Langley G., Evans T., Holgate S.T., Jones A. (2007). Replacing animal experiments: Choices, chances and challenges. BioEssays.

[78] Sena E.S., van der Worp H.B., Bath P.M., Howells D.W., Macleod M.R. (2010). Publication bias in reports of animal stroke studies leads to major overstatement of efficacy. PLoS Biol..

[79] Upadhyay R.K. (2014). Drug delivery systems, cns protection, and the blood brain barrier. Biomed. Res. Int..

[80] Alavijeh M.S., Chishty M., Qaiser M.Z., Palmer A.M. (2005). Drug metabolism and pharmacokinetics, the blood-brain barrier, and central nervous system drug discovery. NeuroRx.

[81] Pardridge W.M. (2005). The blood-brain barrier: Bottleneck in brain drug development. NeuroRx.

[82] Sanchez-Covarrubias L., Slosky L.M., Thompson B.J., Davis T.P., Ronaldson P.T. (2014). Transporters at cns barrier sites: Obstacles or opportunities for drug delivery?. Curr. Pharm. Des..

[83] Ek C.J., D'Angelo B., Baburamani A.A., Lehner C., Leverin A.L., Smith P.L., Nilsson H., Svedin P., Hagberg H., Mallard C. (2015). Brain barrier properties and cerebral blood flow in neonatal mice exposed to cerebral hypoxia-ischemia. J. Cereb. Blood Flow Metab..

[84] Allegaert K., van den Anker J.N. (2014). Clinical pharmacology in neonates: Small size, huge variability. Neonatology.

[85] Turner M.A. (2011). Neonatal drug development. Early Hum. Dev..

[86] Dabliz R., Levine S. (2012). Medication safety in neonates. Am. J. Perinatol..

[87] Du W., Lehr V.T., Lieh-Lai M., Koo W., Ward R.M., Rieder M.J., van den Anker J.N., Reeves J.H., Mathew M., Lulic-Botica M. (2013). An algorithm to detect adverse drug reactions in the neonatal intensive care unit. J. Clin. Pharmacol..

[88] Tuleu C. (2012). 'Formulating better medicines for children'—Still paving the road. Int. J. Pharm..

[89] Van den Anker J., Allegaert K. (2012). Clinical pharmacology in neonates and young infants: The benefit of a population-tailored approach. Exp. Rev. Clin. Pharmacol..

[90] Choonara I. (2008). Who wants safer medicines for children. Arch. Dis. Child..

[91] Salunke S., Giacoia G., Tuleu C. (2012). The step (safety and toxicity of excipients for paediatrics) database. Part 1—A need assessment study. Int. J. Pharm..

[92] Nahata M.C. (2009). Safety of “inert” additives or excipients in paediatric medicines. Arch. Dis. child. Fetal Neonatal Ed..

[93] Turner M.A., Storme T. (2012). European study for neonatal excipient exposure (esnee). Eur. J. Hosp. Pharm..

[94] Johnston M.V., Fatemi A., Wilson M.A., Northington F. (2011). Treatment advances in neonatal neuroprotection and neurointensive care. Lancet Neurol..

[95] O'Regan A., Berman J.S. (2000). Osteopontin: A key cytokine in cell-mediated and granulomatous inflammation. Int. J. Exp. Pathol..

[96] Chabot S., Williams G., Yong V.W. (1997). Microglial production of tnf-alpha is induced by activated T lymphocytes. Involvement of vla-4 and inhibition by interferonbeta-1b. J. Clin. Investig..

[97] Dhanasekaran D.N., Reddy E.P. (2008). Jnk signaling in apoptosis. Oncogene.

[98] Nijboer C.H., Heijnen C.J., Groenendaal F., van Bel F., Kavelaars A. (2009). Alternate pathways preserve tumor necrosis factor-alpha production after nuclear factor-kappab inhibition in neonatal cerebral hypoxia-ischemia. Stroke.

[99] Nijboer C.H., Bonestroo H.J., Zijlstra J., Kavelaars A., Heijnen C.J. (2013). Mitochondrial jnk phosphorylation as a novel therapeutic target to inhibit neuroinflammation and apoptosis after neonatal ischemic brain damage. Neurobiol. Dis..

[100] Meyn D.F., Ness J., Ambalavanan N., Carlo W.A. (2010). Prophylactic phenobarbital and whole-body cooling for neonatal hypoxic-ischemic encephalopathy. J. Pediatr..

[101] Alonso-Alconada D., Alvarez A., Arteaga O., Martinez-Ibarguen A., Hilario E. (2013). Neuroprotective effect of melatonin: A novel therapy against perinatal hypoxia-ischemia. Int. J. Mol. Sci..

[102] Carloni S., Perrone S., Buonocore G., Longini M., Proietti F., Balduini W. (2008). Melatonin protects from the long-term consequences of a neonatal hypoxic-ischemic brain injury in rats. J. Pineal. Res..

[103] Robertson N.J., Faulkner S., Fleiss B., Bainbridge A., Andorka C., Price D., Powell E., Lecky-Thompson L., Thei L., Chandrasekaran M. (2013). Melatonin augments hypothermic neuroprotection in a perinatal asphyxia model. Brain.

[104] Aly H., Elmahdy H., El-Dib M., Rowisha M., Awny M., El-Gohary T., Elbatch M., Hamisa M., El-Mashad A.R. (2015). Melatonin use for neuroprotection in perinatal asphyxia: A randomized controlled pilot study. J. Perinatol..

[105] Takizawa Y., Miyazawa T., Nonoyama S., Goto Y., Itoh M. (2009). Edaravone inhibits DNA peroxidation and neuronal cell death in neonatal hypoxic-ischemic encephalopathy model rat. Pediatr. Res..

[106] Noor J.I., Ueda Y., Ikeda T., Ikenoue T. (2007). Edaravone inhibits lipid peroxidation in neonatal hypoxic-ischemic rats: An *in vivo* microdialysis study. Neurosci. Lett..

[107] Ni X., Yang Z.J., Carter E.L., Martin L.J., Koehler R.C. (2011). Striatal neuroprotection from neonatal hypoxia-ischemia in piglets by antioxidant treatment with euk-134 or edaravone. Dev. Neurosci..

[108] Villa P., Bigini P., Mennini T., Agnello D., Laragione T., Cagnotto A., Viviani B., Marinovich M., Cerami A., Coleman T.R. (2003). Erythropoietin selectively attenuates cytokine production and inflammation in cerebral ischemia by targeting neuronal apoptosis. J. Exp. Med..

[109] Wang L., Zhang Z., Wang Y., Zhang R., Chopp M. (2004). Treatment of stroke with erythropoietin enhances neurogenesis and angiogenesis and improves neurological function in rats. Stroke.

[110] Juul S.E. (2013). Hypothermia plus erythropoietin for neonatal neuroprotection? Commentary on Fan *et al.* and Fang *et al.*. Pediatr. Res..

[111] Bernaudin M., Marti H.H., Roussel S., Divoux D., Nouvelot A., MacKenzie E.T., Petit E. (1999). A potential role for erythropoietin in focal permanent cerebral ischemia in mice. J. Cereb. Blood Flow Metab..

[112] Mu D., Chang Y.S., Vexler Z.S., Ferriero D.M. (2005). Hypoxia-inducible factor 1alpha and erythropoietin upregulation with deferoxamine salvage after neonatal stroke. Exp. Neurol..

[113] Palmer C., Towfighi J., Roberts R.L., Heitjan D.F. (1993). Allopurinol administered after inducing hypoxia-ischemia reduces brain injury in 7-day-old rats. Pediatr. Res..

[114] Kaandorp J.J., Benders M.J., Schuit E., Rademaker C.M., Oudijk M.A., Porath M.M., Oetomo S.B., Wouters M.G., van Elburg R.M., Franssen M.T. (2015). Maternal allopurinol administration during suspected fetal hypoxia: A novel neuroprotective intervention? A multicentre randomised placebo controlled trial. Arch. Dis. Child. Fetal Neonatal Ed..

[115] David H.N., Haelewyn B., Rouillon C., Lecoq M., Chazalviel L., Apiou G., Risso J.J., Lemaire M., Abraini J.H. (2008). Neuroprotective effects of xenon: A therapeutic window of opportunity in rats subjected to transient cerebral ischemia. FASEB J..

[116] Ma D., Hossain M., Chow A., Arshad M., Battson R.M., Sanders R.D., Mehmet H., Edwards A.D., Franks N.P., Maze M. (2005). Xenon and hypothermia combine to provide neuroprotection from neonatal asphyxia. Ann. Neurol..

[117] Follett P.L., Deng W., Dai W., Talos D.M., Massillon L.J., Rosenberg P.A., Volpe J.J., Jensen F.E. (2004). Glutamate receptor-mediated oligodendrocyte toxicity in periventricular leukomalacia: A protective role for topiramate. J. Neurosci..

[118] Kaminski R.M., Banerjee M., Rogawski M.A. (2004). Topiramate selectively protects against seizures induced by atpa, a glur5 kainate receptor agonist. Neuropharmacology.

[119] Koh S., Tibayan F.D., Simpson J.N., Jensen F.E. (2004). Nbqx or topiramate treatment after perinatal hypoxia-induced seizures prevents later increases in seizure-induced neuronal injury. Epilepsia.

[120] Zona C., Ciotti M.T., Avoli M. (1997). Topiramate attenuates voltage-gated sodium currents in rat cerebellar granule cells. Neurosci. Lett..

[121] Costa C., Martella G., Picconi B., Prosperetti C., Pisani A., di Filippo M., Pisani F., Bernardi G., Calabresi P. (2006). Multiple mechanisms underlying the neuroprotective effects of antiepileptic drugs against *in vitro* ischemia. Stroke.

[122] Dodgson S.J., Shank R.P., Maryanoff B.E. (2000). Topiramate as an inhibitor of carbonic anhydrase isoenzymes. Epilepsia.

[123] Kudin A.P., Debska-Vielhaber G., Vielhaber S., Elger C.E., Kunz W.S. (2004). The mechanism of neuroprotection by topiramate in an animal model of epilepsy. Epilepsia.

[124] Ozyener F., Cetinkaya M., Alkan T., Goren B., Kafa I.M., Kurt M.A., Koksal N. (2012). Neuroprotective effects of melatonin administered alone or in combination with topiramate in neonatal hypoxic-ischemic rat model. Restor. Neurol. Neurosci..

[125] Noh M.R., Kim S.K., Sun W., Park S.K., Choi H.C., Lim J.H., Kim I.H., Kim H.J., Kim H., Eun B.L. (2006). Neuroprotective effect of topiramate on hypoxic ischemic brain injury in neonatal rats. Exp. Neurol..

[126] Galinsky R., Bennet L., Groenendaal F., Lear C.A., Tan S., van Bel F., Juul S.E., Robertson N.J., Mallard C., Gunn A.J. (2014). Magnesium is not consistently neuroprotective for perinatal hypoxia-ischemia in term-equivalent models in preclinical studies: A systematic review. Dev. Neurosci..

[127] Tagin M., Shah P.S., Lee K.S. (2013). Magnesium for newborns with hypoxic-ischemic encephalopathy: A systematic review and meta-analysis. J. Perinatol..

[128] Diamandis T., Borlongan C.V. (2015). One, two, three steps toward cell therapy for stroke. Stroke.

[129] Rodriguez-Gomez J.A., Lu J.Q., Velasco I., Rivera S., Zoghbi S.S., Liow J.S., Musachio J.L., Chin F.T., Toyama H., Seidel J. (2007). Persistent dopamine functions of neurons derived from embryonic stem cells in a rodent model of parkinson disease. Stem Cells.

[130] Dimos J.T., Rodolfa K.T., Niakan K.K., Weisenthal L.M., Mitsumoto H., Chung W., Croft G.F., Saphier G., Leibel R., Goland R. (2008). Induced pluripotent stem cells generated from patients with als can be differentiated into motor neurons. Science.

[131] Roy N.S., Cleren C., Singh S.K., Yang L., Beal M.F., Goldman S.A. (2006). Functional engraftment of human es cell-derived dopaminergic neurons enriched by coculture with telomerase-immortalized midbrain astrocytes. Nat. Med..

[132] Bliss T., Guzman R., Daadi M., Steinberg G.K. (2007). Cell transplantation therapy for stroke. Stroke.

[133] Liao Y., Cotten M., Tan S., Kurtzberg J., Cairo M.S. (2013). Rescuing the neonatal brain from hypoxic injury with autologous cord blood. Bone Marrow Transplant..

[134] Denhardt D.T., Noda M., O'Regan A.W., Pavlin D., Berman J.S. (2001). Osteopontin as a means to cope with environmental insults: Regulation of inflammation, tissue remodeling, and cell survival. J. Clin. Investig..

[135] Mazzali M., Kipari T., Ophascharoensuk V., Wesson J.A., Johnson R., Hughes J. (2002). Osteopontin-a molecule for all seasons. QJM: Int. J. Med..

[136] van Velthoven C.T., Heijnen C.J., van Bel F., Kavelaars A. (2011). Osteopontin enhances endogenous repair after neonatal hypoxic-ischemic brain injury. Stroke.

[137] Chen W., Ma Q., Suzuki H., Hartman R., Tang J., Zhang J.H. (2011). Osteopontin reduced hypoxia-ischemia neonatal brain injury by suppression of apoptosis in a rat pup model. Stroke.

[138] Bonestroo H.J., Nijboer C.H., van Velthoven C.T., van Bel F., Heijnen C.J. (2015). The neonatal brain is not protected by osteopontin peptide treatment after hypoxia-ischemia. Dev. Neurosci..

[139] Veldhuis W.B., Floris S., van der Meide P.H., Vos I.M., de Vries H.E., Dijkstra C.D., Bar P.R., Nicolay K. (2003). Interferon-beta prevents cytokine-induced neutrophil infiltration and attenuates blood-brain barrier disruption. J. Cereb. Blood Flow Metab..

[140] Maier C.M., Yu F., Nishi T., Lathrop S.J., Chan P.H. (2006). Interferon-beta fails to protect in a model of transient focal stroke. Stroke.

[141] Bogoyevitch M.A., Boehm I., Oakley A., Ketterman A.J., Barr R.K. (2004). Targeting the jnk mapk cascade for inhibition: Basic science and therapeutic potential. Biochim. Biophys. Acta Prot. Proteom..

[142] Karin M., Gallagher E. (2005). From jnk to pay dirt: Jun kinases, their biochemistry, physiology and clinical importance. IUBMB Life.

[143] Nijboer C.H., van der Kooij M.A., van Bel F., Ohl F., Heijnen C.J., Kavelaars A. (2010). Inhibition of the jnk/ap-1 pathway reduces neuronal death and improves behavioral outcome after neonatal hypoxic-ischemic brain injury. Brain Behav. Immun..

[144] Kaandorp J.J., van den Broek M.P., Benders M.J., Oudijk M.A., Porath M.M., Bambang Oetomo S., Wouters M.G., van Elburg R., Franssen M.T., Bos A.F. (2014). Rapid target allopurinol concentrations in the hypoxic fetus after maternal administration during labour. Arch. Dis. Child. Fetal Aneonatal Ed..

[145] Merchant N.M., Azzopardi D.V., Hawwa A.F., McElnay J.C., Middleton B., Arendt J., Arichi T., Gressens P., Edwards A.D. (2013). Pharmacokinetics of melatonin in preterm infants. Br. J. Clin. Pharmacol..

[146] Shea K.L., Palanisamy A. (2015). What can you do to protect the newborn brain?. Curr. Opin. Anaesthesiol..

[147] Noor J.I., Ikeda T., Mishima K., Aoo N., Ohta S., Egashira N., Iwasaki K., Fujiwara M., Ikenoue T. (2005). Short-term administration of a new free radical scavenger, edaravone, is more effective than its long-term administration for the treatment of neonatal hypoxic-ischemic encephalopathy. Stroke.

[148] Juul S.E., Yachnis A.T., Rojiani A.M., Christensen R.D. (1999). Immunohistochemical localization of erythropoietin and its receptor in the developing human brain. Pediatr. Dev. Pathol..

[149] Juul S.E., McPherson R.J., Farrell F.X., Jolliffe L., Ness D.J., Gleason C.A. (2004). Erytropoietin concentrations in cerebrospinal fluid of nonhuman primates and fetal sheep following high-dose recombinant erythropoietin. Neonatology.

[150] Zhu C., Kang W., Xu F., Cheng X., Zhang Z., Jia L., Ji L., Guo X., Xiong H., Simbruner G. (2009). Erythropoietin improved neurologic outcomes in newborns with hypoxic-ischemic encephalopathy. Pediatrics.

[151] Plateel M., Teissier E., Cecchelli R. (1997). Hypoxia dramatically increases the nonspecific transport of blood-borne proteins to the brain. J. Neurochem..

[152] Statler P.A., McPherson R.J., Bauer L.A., Kellert B.A., Juul S.E. (2007). Pharmacokinetics of high-dose recombinant erythropoietin in plasma and brain of neonatal rats. Pediatr. Res..

[153] Xenocostas A., Cheung W.K., Farrell F., Zakszewski C., Kelley M., Lutynski A., Crump M., Lipton J.H., Kiss T.L., Lau C.Y. (2005). The pharmacokinetics of erythropoietin in the cerebrospinal fluid after intravenous administration of recombinant human erythropoietin. Eur J. Clin. Pharmacol..

[154] Wu Y.W., Bauer L.A., Ballard R.A., Ferriero D.M., Glidden D.V., Mayock D.E., Chang T., Durand D.J., Song D., Bonifacio S.L. (2012). Erythropoietin for neuroprotection in neonatal encephalopathy: Safety and pharmacokinetics. Pediatrics.

[155] Kumral A., Uysal N., Tugyan K., Sonmez A., Yilmaz O., Gokmen N., Kiray M., Genc S., Duman N., Koroglu T.F. (2004). Erythropoietin improves long-term spatial memory deficits and brain injury following neonatal hypoxia-ischemia in rats. Behav. Brain Res..

[156] Gonzalez F.F., Abel R., Almli C.R., Mu D., Wendland M., Ferriero D.M. (2009). Erythropoietin sustains cognitive function and brain volume after neonatal stroke. Dev. Neurosci..

[157] Rogers E.E., Bonifacio S.L., Glass H.C., Juul S.E., Chang T., Mayock D.E., Durand D.J., Song D., Barkovich A.J., Ballard R.A. (2014). Erythropoietin and hypothermia for hypoxic-ischemic encephalopathy. Pediatr. Neurol..

[158] Shankaran S. (2014). Outcomes of hypoxic-ischemic encephalopathy in neonates treated with hypothermia. Clin. Perinatol..

[159] Elmahdy H., El-Mashad A.R., El-Bahrawy H., El-Gohary T., El-Barbary A., Aly H. (2010). Human recombinant erythropoietin in asphyxia neonatorum: Pilot trial. Pediatrics.

[160] Yin D., Kawabata H., Tcherniamtchouk O., Huynh T., Black K.L., Koeffler H.P. (2007). Glioblastoma multiforme cells: Expression of erythropoietin receptor and response to erythropoietin. Int. J. Oncol..

[161] Digicaylioglu M., Lipton S.A. (2001). Erythropoietin-mediated neuroprotection involves cross-talk between jak2 and nf-kappab signalling cascades. Nature.

[162] Lee S.T., Chu K., Sinn D.I., Jung K.H., Kim E.H., Kim S.J., Kim J.M., Ko S.Y., Kim M., Roh J.K. (2006). Erythropoietin reduces perihematomal inflammation and cell death with enos and stat3 activations in experimental intracerebral hemorrhage. J. Neurochem..

[163] Park M.H., Lee S.M., Lee J.W., Son D.J., Moon D.C., Yoon D.Y., Hong J.T. (2006). Erk-mediated production of neurotrophic factors by astrocytes promotes neuronal stem cell differentiation by erythropoietin. Biochem. Biophys. Res. Commun..

[164] Peeters-Scholte C., Braun K., Koster J., Kops N., Blomgren K., Buonocore G., van Buul-Offers S., Hagberg H., Nicolay K., van Bel F. (2003). Effects of allopurinol and deferoxamine on reperfusion injury of the brain in newborn piglets after neonatal hypoxia-ischemia. Pediatr. Res..

[165] Benders M.J., Bos A.F., Rademaker C.M., Rijken M., Torrance H.L., Groenendaal F., van Bel F. (2006). Early postnatal allopurinol does not improve short term outcome after severe birth asphyxia. Arch. Dis. Child. Fetal Neonatal Ed..

[166] Kaandorp J.J., van Bel F., Veen S., Derks J.B., Groenendaal F., Rijken M., Roze E., Venema M.M., Rademaker C.M., Bos A.F. (2012). Long-term neuroprotective effects of allopurinol after moderate perinatal asphyxia: Follow-up of two randomised controlled trials. Arch. Dis. Child. Fetal Neonatal Ed..

[167] Dworschak M. (2008). Pharmacologic neuroprotection-is xenon the light at the end of the tunnel?. Crit. Care Med..

[168] Istaphanous G.K., Loepke A.W. (2009). General anesthetics and the developing brain. Curr. Opin. Anaesthesiol..

[169] Hobbs C., Thoresen M., Tucker A., Aquilina K., Chakkarapani E., Dingley J. (2008). Xenon and hypothermia combine additively, offering long-term functional and histopathologic neuroprotection after neonatal hypoxia/ischemia. Stroke.

[170] Thoresen M., Hobbs C.E., Wood T., Chakkarapani E., Dingley J. (2009). Cooling combined with immediate or delayed xenon inhalation provides equivalent long-term neuroprotection after neonatal hypoxia-ischemia. J. Cereb. Blood Flow Metab..

[171] Dingley J., Tooley J., Liu X., Scull-Brown E., Elstad M., Chakkarapani E., Sabir H., Thoresen M. (2014). Xenon ventilation during therapeutic hypothermia in neonatal encephalopathy: A feasibility study. Pediatrics.

[172] Robertson N.J., Tan S., Groenendaal F., van Bel F., Juul S.E., Bennet L., Derrick M., Back S.A., Valdez R.C., Northington F. (2012). Which neuroprotective agents are ready for bench to bedside translation in the newborn infant?. J. Pediatr..

[173] Cilio M.R., Ferriero D.M. (2010). Synergistic neuroprotective therapies with hypothermia. Semin. Fetal Neonatal Med..

[174] Guerrini R., Parmeggiani L. (2006). Topiramate and its clinical applications in epilepsy. Expert Opin. Pharmacother..

[175] Shank R.P., Gardocki J.F., Streeter A.J., Maryanoff B.E. (2000). An overview of the preclinical aspects of topiramate: Pharmacology, pharmacokinetics, and mechanism of action. Epilepsia.

[176] Angehagen M., Ronnback L., Hansson E., Ben-Menachem E. (2005). Topiramate reduces ampa-induced Ca^2+^ transients and inhibits glur1 subunit phosphorylation in astrocytes from primary cultures. J. Neurochem..

[177] Sfaello I., Baud O., Arzimanoglou A., Gressens P. (2005). Topiramate prevents excitotoxic damage in the newborn rodent brain. Neurobiol. Dis..

[178] Zeevalk G.D., Nicklas W.J. (1992). Evidence that the loss of the voltage-dependent Mg^2+^ block at the n-methyl-d-aspartate receptor underlies receptor activation during inhibition of neuronal metabolism. J. Neurochem..

[179] Sugimoto J., Romani A.M., Valentin-Torres A.M., Luciano A.A., Ramirez Kitchen C.M., Funderburg N., Mesiano S., Bernstein H.B. (2012). Magnesium decreases inflammatory cytokine production: A novel innate immunomodulatory mechanism. J. Immunol..

[180] Hoffman D.J., Marro P.J., McGowan J.E., Mishra O.P., Delivoria-Papadopoulos M. (1994). Protective effect of mgso4 infusion on nmda receptor binding characteristics during cerebral cortical hypoxia in the newborn piglet. Brain Res..

[181] Shokry M., Elsedfy G.O., Bassiouny M.M., Anmin M., Abozid H. (2010). Effects of antenatal magnesium sulfate therapy on cerebral and systemic hemodynamics in preterm newborns. Acta Obstet. Gynecol. Scand..

[182] de Haan H.H., Gunn A.J., Williams C.E., Heymann M.A., Gluckman P.D. (1997). Magnesium sulfate therapy during asphyxia in near-term fetal lambs does not compromise the fetus but does not reduce cerebral injury. Am. J. Obstet. Gynecol..

[183] Penrice J., Amess P.N., Punwani S., Wylezinska M., Tyszczuk L., D'Souza P., Edwards A.D., Cady E.B., Wyatt J.S., Reynolds E.O. (1997). Magnesium sulfate after transient hypoxia-ischemia fails to prevent delayed cerebral energy failure in the newborn piglet. Pediatr. Res..

[184] Greenwood K., Cox P., Mehmet H., Penrice J., Amess P.N., Cady E.B., Wyatt J.S., Edwards A.D. (2000). Magnesium sulfate treatment after transient hypoxia-ischemia in the newborn piglet does not protect against cerebral damage. Pediatr. Res..

[185] Schira J., Gasis M., Estrada V., Hendricks M., Schmitz C., Trapp T., Kruse F., Kogler G., Wernet P., Hartung H.P. (2012). Significant clinical, neuropathological and behavioural recovery from acute spinal cord trauma by transplantation of a well-defined somatic stem cell from human umbilical cord blood. Brain.

[186] Escolar M.L., Poe M.D., Provenzale J.M., Richards K.C., Allison J., Wood S., Wenger D.A., Pietryga D., Wall D., Champagne M. (2005). Transplantation of umbilical-cord blood in babies with infantile krabbe's disease. N. Engl. J. Med..

[187] Sun J., Allison J., McLaughlin C., Sledge L., Waters-Pick B., Wease S., Kurtzberg J. (2010). Differences in quality between privately and publicly banked umbilical cord blood units: A pilot study of autologous cord blood infusion in children with acquired neurologic disorders. Transfusion.

[188] Cotten C.M., Murtha A.P., Goldberg R.N., Grotegut C.A., Smith P.B., Goldstein R.F., Fisher K.A., Gustafson K.E., Waters-Pick B., Swamy G.K. (2014). Feasibility of autologous cord blood cells for infants with hypoxic-ischemic encephalopathy. J. Pediatr..

[189] Borlongan C.V., Weiss M.D. (2011). Baby steps: A giant leap for cell therapy in neonatal brain injury. Pediatr. Res..

[190] Gonzales-Portillo G.S., Reyes S., Aguirre D., Pabon M.M., Borlongan C.V. (2014). Stem cell therapy for neonatal hypoxic-ischemic encephalopathy. Front. Neurol..

[191] Katsuragi S., Ikeda T., Date I., Shingo T., Yasuhara T., Mishima K., Aoo N., Harada K., Egashira N., Iwasaki K. (2005). Implantation of encapsulated glial cell line-derived neurotrophic factor-secreting cells prevents long-lasting learning impairment following neonatal hypoxic-ischemic brain insult in rats. Am. J. Obstet. Gynecol..

[192] Zalewska T., Jaworska J., Ziemka-Nalecz M. (2015). Current and experimental pharmacological approaches in neonatal hypoxic- ischemic encephalopathy. Curr. Pharm. Des..

[193] Douglas-Escobar M., Weiss M.D. (2012). Biomarkers of hypoxic-ischemic encephalopathy in newborns. Front. Neurol..

[194] Bennet L., Booth L., Gunn A.J. (2010). Potential biomarkers for hypoxic-ischemic encephalopathy. Semin. Fetal Neonatal Med..

[195] Meier C., Middelanis J., Wasielewski B., Neuhoff S., Roth-Haerer A., Gantert M., Dinse H.R., Dermietzel R., Jensen A. (2006). Spastic paresis after perinatal brain damage in rats is reduced by human cord blood mononuclear cells. Pediatr. Res..

[196] Geissler M., Dinse H.R., Neuhoff S., Kreikemeier K., Meier C. (2011). Human umbilical cord blood cells restore brain damage induced changes in rat somatosensory cortex. PLoS ONE.

[197] Pimentel-Coelho P.M., Magalhães E.S., Lopes L.M., deAzevedo L.C., Santiago M.F., Mendez-Otero R. (2010). Human cord blood transplantation in a neonatal rat model of hypoxic-ischemic brain damage: Functional outcome related to neuroprotection in the striatum. Stem Cells Dev..

[198] Yasuhara T., Hara K., Maki M., Xu L., Yu G., Ali M.M., Masuda T., Yu S.J., Bae E.K., Hayashi T. (2010). Mannitol facilitates neurotrophic factor up-regulation and behavioural recovery in neonatal hypoxic-ischaemic rats with human umbilical cord blood grafts. J. Cell. Mol. Med..

[199] De Paula S., Vitola A.S., Greggio S., de Paula D., Mello P.B., Lubianca J.M., Xavier L.L., Fiori H.H., Dacosta J.C. (2009). Hemispheric brain injury and behavioral deficits induced by severe neonatal hypoxia-ischemia in rats are not attenuated by intravenous administration of human umbilical cord blood cells. Pediatr. Res..

[200] Greggio S., de Paula S., Azevedo P.N., Venturin G.T., Dacosta J.C. (2014). Intra-arterial transplantation of human umbilical cord blood mononuclear cells in neonatal hypoxic-ischemic rats. Life Sci..

[201] Wang X., Zhao Y., Wang X. (2014). Umbilical cord blood cells regulate the differentiation of endogenous neural stem cells in hypoxic ischemic neonatal rats via the hedgehog signaling pathway. Brain Res..

[202] Yasuhara T., Matsukawa N., Yu G., Xu L., Mays R.W., Kovach J., Deans R.J., Hess D.C., Carroll J.E., Borlongan C.V. (2006). Behavioral and histological characterization of intrahippocampal grafts of human bone marrow-derived multipotent progenitor cells in neonatal rats with hypoxic-ischemic injury. Cell. Transplant..

[203] Yasuhara T., Hara K., Maki M., Mays R.W., Deans R.J., Hess D.C., Carroll J.E., Borlongan C.V. (2008). Intravenous grafts recapitulate the neurorestoration afforded by intracerebrally delivered multipotent adult progenitor cells in neonatal hypoxic-ischemic rats. J. Cereb. Blood Flow Metab..

[204] Van Velthoven C.T.J., Kavelaars A., van Bel F., Heijnen C.J. (2010). Repeated mesenchymal stem cell treatment after neonatal hypoxia-ischemia has distinct effects on formation and maturation of new neurons and oligodendrocytes leading to restoration of damage, corticospinal motor tract activity, and sensorimotor function. J. Neurosci..

[205] Van Velthoven C.T.J., Kavelaars A., van Bel F., Heijnen C.J. (2010). Nasal administration of stem cells: A promising novel route to treat neonatal ischemic brain damage. Pediatr. Res..

[206] Donega V., Nijboer C.H., van Tilborg G., Dijkhuizen R.M., Kavelaars A., Heijnen C.J. (2014). Intranasally administered mesenchymal stem cells promote a regenerative niche for repair of neonatal ischemic brain injury. Exp. Neurol..

[207] Van Velthoven C.T., Braccioli L., Willemen H.L., Kavelaars A., Heijnen C.J. (2014). Therapeutic potential of genetically modified mesenchymal stem cells after neonatal hypoxic-ischemic brain damage. Mol. Ther..

[208] Liu Y., Jiang X., Zhang X., Chen R., Sun T., Fok K.L., Dong J., Tsang L.L., Yi S., Ruan Y. (2011). Dedifferentiation-reprogrammed mesenchymal stem cells with improved therapeutic potential. Stem Cells.

[209] Kim E.S., Ahn S.Y., Im G.H., Sung D.K., Park Y.R., Choi S.H., Choi S.J., Chang Y.S., Oh W., Lee J.H. (2012). Human umbilical cord blood-derived mesenchymal stem cell transplantation attenuates severe brain injury by permanent middle cerebral artery occlusion in newborn rats. Pediatr. Res..

[210] Park W.S., Sung S.I., Ahn S.Y., Yoo H.S., Sung D.K., Im G.H., Choi S.J., Chang Y.S. (2015). Hypothermia augments neuroprotective activity of mesenchymal stem cells for neonatal hypoxic-ischemic encephalopathy. PLoS ONE.

[211] Xia G., Hong X., Chen X., Lan F., Zhang G., Liao L. (2010). Intracerebral transplantation of mesenchymal stem cells derived from human umbilical cord blood alleviates hypoxic ischemic brain injury in rat neonates. J. Perinat. Med..

[212] Zhang X., Zhang Q., Li W., Nie D., Chen W., Xu C., Yi X., Shi J., Tian M., Qin J. (2014). Therapeutic effect of human umbilical cord mesenchymal stem cells on neonatal rat hypoxic-ischemic encephalopathy. J. Neurosci. Res..

[213] Lee J.A., Kim B.I., Jo C.H., Choi C.W., Kim E.-K., Kim H.-S., Yoon K.-S., Choi J.-H. (2010). Mesenchymal stem-cell transplantation for hypoxic-ischemic brain injury in neonatal rat model. Pediatr. Res..

[214] Yamagata M., Yamamoto A., Kako E., Kaneko N., Matsubara K., Sakai K., Sawamoto K., Ueda M. (2013). Human dental pulp-derived stem cells protect against hypoxic-ischemic brain injury in neonatal mice. Stroke.

[215] Fang C., Yang Y., Wang Q., Yao Y., Zhang X., He X. (2013). Intraventricular injection of human dental pulp stem cells improves hypoxic-ischemic brain damage in neonatal rats. PLoS ONE.

[216] Park K.I., Teng Y.D., Snyder E.Y. (2002). The injured brain interacts reciprocally with neural stem cells supported by scaffolds to reconstitute lost tissue. Nat. Biotechnol..

[217] Park K.I., Himes B.T., Stieg P.E., Tessler A., Fischer I., Snyder E.Y. (2006). Neural stem cells may be uniquely suited for combined gene therapy and cell replacement: Evidence from engraftment of Neurotrophin-3-expressing stem cells in hypoxic-ischemic brain injury. Exp. Neurol..

[218] Daadi M.M., Davis A.S., Arac A., Li Z., Maag A.-L., Bhatnagar R., Jiang K., Sun G., Wu J.C., Steinberg G.K. (2010). Human neural stem cell grafts modify microglial response and enhance axonal sprouting in neonatal hypoxic-ischemic brain injury. Stroke.

[219] Ashwal S., Ghosh N., Turenius C.I., Dulcich M., Denham C.M., Tone B., Hartman R., Snyder E.Y., Obenaus A. (2014). Reparative effects of neural stem cells in neonatal rats with hypoxic-ischemic injury are not influenced by host sex. Pediatr. Res..

[220] Chen L.-X., Ma S.-M., Zhang P., Fan Z.-C., Xiong M., Cheng G.-Q., Yang Y., Qiu Z.-L., Zhou W.-H., Li J. (2015). Neuroprotective effects of oligodendrocyte progenitor cell transplantation in premature rat brain following hypoxic-ischemic injury. PLoS ONE.

